# Characterization of *Pantoea ananatis* from rice planthoppers reveals a clade of rice-associated *P. ananatis* undergoing genome reduction

**DOI:** 10.1099/mgen.0.000907

**Published:** 2022-12-05

**Authors:** Xiao-Li Bing, Yu-Ying Wan, Huan-Huan Liu, Rui Ji, Dian-Shu Zhao, Yue-Di Niu, Tong-Pu Li, Xiao-Yue Hong

**Affiliations:** ^1^​ Department of Entomology, Nanjing Agricultural University, Nanjing, Jiangsu 210095, PR China; ^2^​ Institute of Plant Protection, Jiangsu Academy of Agricultural Sciences, Nanjing, Jiangsu 210014, PR China

**Keywords:** comparative genomics, *Laodelphax striatellus*, *Pantoea ananatis*, pathogen, rice, T6SS

## Abstract

*
Pantoea ananatis* is a bacterium that is found in many agronomic crops and agricultural pests. Here, we isolated a *P. ananatis* strain (Lstr) from the rice planthopper *Laodelphax striatellus*, a notorious pest that feeds on rice plant sap and transmits rice viruses, in order to examine its genome and biology. *P. ananatis* Lstr is an insect symbiont that is pathogenic to the host insect and appears to mostly inhabit the gut. Its pathogenicity thus raises the possibility of using the Lstr strain as a biological agent. To this end, we analysed the genome of the Lstr strain and compared it with the genomes of other *Pantoea* species. Our analysis of these genomes shows that *P. ananatis* can be divided into two mono-phylogenetic clades (clades one and two). The Lstr strain belongs to clade two and is grouped with *P. ananatis* strains that were isolated from rice or rice-associated samples. A comparative genomic analysis shows that clade two differs from clade one in many genomic characteristics including genome structures, mobile elements, and categories of coding proteins. The genomes of clade two *P *. *ananatis* are significantly smaller, have much fewer coding sequences but more pseudogenes than those of clade one, suggesting that clade two species are at the early stage of genome reduction. On the other hand, *P. ananatis* has a type VI secretion system that is highly variable but cannot be separated by clades. These results clarify our understanding of *P. ananatis *’ phylogenetic diversity and provide clues to the interactions between *P. ananatis *, host insect, and plant that may lead to advances in rice protection and pest control.

## Data Summary

Raw reads for *Pantoea ananatis* Lstr are available through NCBI under BioProject accession PRJNA660206. One supplementary figure file and eight supplementary tables are available with the online version of this article. All supplementary files are available from Zenodo data repository under the DOI 10.5281/zenodo.7040480.

**Impact Statement**

Symbionts and pathogens are ubiquitous in nature and have complex interactions with hosts. During their long-time co-evolution, many genomes of recently evolved symbionts and pathogens contain characteristics of genome reduction. *Pantoea ananatis* is a cosmopolitan aerobic or facultatively anaerobic bacterium that is found in many agronomic crops and agricultural pests. This study explored the biology and genomic features of a *P. ananatis* strain (Lstr) isolated from the rice planthopper, a notorious rice pest. *P. ananatis* Lstr is an insect gut symbiont that is pathogenic to the host insect. Analysis of genomes of Lstr and other *Pantoea* species shows that *P. ananatis* can be divided into two mono-phylogenetic clades (clades one and two). Clade two contains the Lstr strain and other *P. ananatis* strains that were isolated mainly from rice or rice-associated samples. Comparative genomic results show that clade two species have undergone a genome reduction. Moreover, *P. ananatis* has a T6SS that is highly variable and many T6SS genes may have been involved in multiple horizontal gene transfers. Our results highlight the complex interactions between *P. ananatis *, host insect, and plant that may lead to advances in rice protection and pest control.

## Introduction

*Pantoea ananatis* (Gammaproteobacteria, Enterobacteriales, *Enterobacteriaceae *) is a cosmopolitan aerobic or facultatively anaerobic bacterium that inhabits a wide variety of environments including soil, rivers, plant materials, insect guts as well as humans [1]. Since its first identification as the causal agent of fruitlet rot of pineapple (*Ananas comosus*) in the Philippines in 1928 [2], *P. ananatis* has been known to cause severe losses of many other agronomic crop and tree species, including maize, onion, *Eucalyptus*, and rice [3–5]. The disease symptoms induced by *P. ananatis* infection include leaf blotch, spot, blight and dieback, stalk, fruit, and bulb rot depending on the specific host (reviewed in [6]). On the other hand, some *P. ananatis* strains benefit plants by promoting their growth [7].

*P. ananatis* strains have also been found in many insects, including mulberry pyralid (*Glyphodes pyloalis*), brown planthopper (*Nilaparvata lugens*), tobacco thrips (*Frankliniella fusca*), cotton fleahopper (*Pseudatomoscelis seriatus*), western corn rootworm (*Diabrotica virgifera virgifera*) and fall armyworm (*Spodoptera frugiperda*) [8–14]. In tobacco thrips, *P. ananatis* was located only in the gut and was horizontally transmitted through thrip faeces [15], although it had no significant effects on fecundity or development [16]. *P. ananatis* isolated from surface-sterilized tobacco thrips induces centre rot of onion [12], indicating thrips function as the vector of pathogenic *P. ananatis* in onion. In addition, *P. ananatis* has developed an endophytic relationship with the cotton fleahopper that resulted in bacterial horizontal transmission when fleahoppers feed on cotton buds [11].

Multiple studies have reported an association between *P. ananatis* and Asian rice *Oryza sativa* [3, 10, 17–22], which feeds almost half of the world’s population. Rice is an ancient crop domesticated about 9000 years ago and now is produced in about 120 countries worldwide. In rice, *P. ananatis* strains can be beneficial by promoting rice growth under saline stress [18], by being strongly antagonistic to rice pathogens [21], and by facilitating rice straw degradation through its lignocellulose degradation capacity [23]. On the other hand, some *P. ananatis* strains are pathogenic as they are the causal agents of rice leaf blight [3, 20], rice grain rot and discolouration [17, 22].

The small brown planthopper *Laodelphax striatellus* (Fallén) (Hemiptera: Delphacidae) is a destructive pest of rice. *L. striatellus* causes direct physiological damage to crops via feeding on plant sap and laying eggs in rice tissues, and causes indirect economic loss by transmitting plant viruses (such as rice stripe virus) [24]. *Pantoea* was detected recently in many *L. striatellus* populations [25]. However, the biology and functions of *Pantoea* in planthoppers were unknown.

Isolation of *P. ananatis* from both plants and animals indicates that it has adapted to a wide range of ecological environments. Genome sequencing can be used to better understand the evolution and functions of *P. ananatis* in general. In a pan-genomic analysis, De Maayer *et al*. [26] found many factors with putative roles in colonization and interaction of *P. ananatis* with plant, insect, and vertebrate hosts. In particular, genomic analysis showed the secretion system genes and mobile genetic elements are different in closely related *P. ananatis* strains found on maize seeds but show different effects on host plants [27]. A pan-genomic analysis revealed a novel series of genetic regions that are correlated with pathogenicity of *P. ananatis* in onion [28]. However, the pathogenicity of *P. ananatis* to insect hosts and the genomic insights of insect-associated *P. ananatis* remain poorly understood.

Here, we studied the localization and pathogenicity of one *P. ananatis* strain (Lstr) that was recently isolated from *L. striatellus* and explored how Lstr is transmitted among insects. In addition, we described the *P. ananatis* Lstr genome and compared it with other *P. ananatis* genomes. Our genomic results show that *P. ananatis* can be grouped into two clades (one and two), which differ not only in phylogeny but also in many other genomic features. The clade two members are closely associated with rice habitats. We found the type VI secretion system (T6SS) that is important for pathogenicity of *P. ananatis* did not cluster according to phylogeny, suggesting that it might have evolved independently.

## Methods

### Maintenance of rice planthoppers, rice, and *P. ananatis*

Small brown planthoppers *L. striatellus* were collected from field rice *Oryza sativa* L. in 2014 in Nanjing, Jiangsu, China. Stock cultures were reared on rice (*Oryza sativa* L. *cv*. Nanjing 3908) seedlings in insect-proof cages placed in growth chambers at 26±2 °C, 50–60% relative humidity and a photoperiod of 16 h/8 h (light/dark). *P. ananatis* Lstr was isolated and identified from surface-sterilized *L. striatellus* adults as described previously [29]. The isolates were stored in 25 % glycerol stocks at −80 °C and maintained on LB agar plates at 30 °C.

### Fluorescence *in situ* hybridization (FISH)

The distribution of *Pantoea* in *L. striatellus* was detected by fluorescence *in situ* hybridization (FISH) with previously described protocols [30]. The *Pantoea* probe, Pan‐rrs-FAM (5′‐TCATCCGATAGTGAGAGGCC‐3′), was designed to target the 16S rRNA gene of *Pantoea *. The specificity of the *Pantoea* probe was checked by ‘probe match’ in RDP 10 [31] and blast in the ‘nr’ database of NCBI (http://blast.ncbi.nlm.nih.gov/Blast.cgi). Two rhodamine-labelled probes W1 (5′-AATCCGGCCGARCCGACCC-3′), and W2 (5′-CTTCTGTGAGTACCGTCATTATC-3′), were used to target the *Wolbachia* 16S rRNA gene [32]. The DNA in the nucleus was stained with 4′,6-diamidino-2-phenylindole (DAPI) (Solarbio, Beijing, China). Stained samples were viewed under a Leica TCS SP8 laser confocal microscope (Leica, Wetzlar, Germany) hosted by the Public Experimental Facility of the College of Plant Protection, Nanjing Agricultural University.

### Diagnostic PCR of microbes

Bacterial infection was confirmed by diagnostic PCR as previously described [33]. Total genomic DNA of planthopper eggs was extracted using the Wizard SV Genomic DNA Purification System (Promega, Fitchburg, WI, USA). The 16S rRNA gene fragment of *Pantoea* which covered four variable regions (V2-V5, 809 bp) was amplified using the primers Pan-rrs-164F (5′- GGCCTCTCACTATCGGATGA −3′) and Pan-rrs-952R (5′- GGCATCTCTGCCAAATTCCG −3′). The presence of *Wolbachia* was screened based on the amplification of a *wsp* fragment with the primers 81F and 691R [34]. PCR analyses were performed using 2×Rapid Taq Master Mix (Vazyme, Nanjing, China) in a Mastercycler nexus PCR Thermo Cycler (Eppendorf, Hamburg, Germany). PCR procedures were an initial step of 95 °C for 3 min, followed by 38 cycles of 95 °C for 15 s, 55 °C for 15 s, and 72 °C for 10 s and a final step of 72 °C for 5 min. Amplified DNA products were electrophoresed on agarose gels and confirmed by Sanger sequencing.

### Bacterial infection

The pathogenicity of *P. ananatis* against rice planthoppers was evaluated with both systemic infection and oral infection. For systemic infection, we pricked *L. striatellus* adults in the thorax with a 0.15 mm needle (Austerlitz Insect Pins, Czech Republic) which was previously dipped in a concentrated bacterial suspension (OD_600_=1, about 10^8^ bacterial cells per millilitre) [29]. The sterile phosphate buffered saline (PBS) solution was used as a control. For oral infections, adult individuals (4 to 8 days old) were starved for 3 h in an empty vial before allowing them to feed on an infection solution containing a suspended culture of bacteria (OD_600_=0.1, 1, 5, or 10 depending on the experiment) and a 2.5 % sucrose solution (v/v=1 : 1) for 12 h. PBS and the sucrose solution were used as controls. After feeding, planthoppers were placed into vials with fresh rice seedlings and were transferred to new rice seedlings every 2 days. The survival of insects was monitored daily for 1 week. Survival curves were analysed with the Cox Proportional Hazards (CoxPH) model with the ‘survival’ package on the statistical software R version 4.0 [35].

### Genome sequencing, assembly, and annotation

We sequenced the genome of *P. ananatis* Lstr aiming to obtain further insights about its pathogenicity. Total DNA was sequenced using the PacBio RS II platform and BGI-500 platform at the Beijing Genomics Institute (BGI, Shenzhen, China). The PacBio reads were self-corrected with the programme Pbdagcon [36]. The bacterial genome was assembled from PacBio and Illumina reads using the programme Celera Assembler 8.3 [37], and was further corrected using GATK 1.6–13 (Genome Analysis Toolkit) [38] and SOAP tool packages (SOAP2, SOAPsnp, and SOAPindel) [39, 40]. Bacterial plasmids were identified by mapping the filtered Illumina reads to the bacterial plasmid database (http://www.ebi.ac.uk/genomes/plasmid.html, last accessed 8 July 2016) using SOAP [41]. One complete circular chromosome and a plasmid were obtained in total.

The genome of *P. ananatis* Lstr was annotated using the NCBI Prokaryotic Genome Annotation Pipeline (PGAP) [42]. The genome size and GC content were calculated with seqkit v0.10.1 [43]. The proteomes were functionally characterized and compared by assigning predicted proteins to the eggNOG, KEGG, and COG databases with eggNOG-mapper v2 [44], BlastKOALA [45] and cdd2cog [46], respectively. The signal peptides and transmembrane domains of proteins were identified with SignalP 4.0 [47] and TMHMM 2.0 [48], respectively. Bacterial secretion system genes were annotated with eggnog, EffectiveDB [44, 49], and blasting *P. anantis* proteomes against a group of characterized T3SS proteins [50]. The insertion sequences were identified based on the ISfinder database [51]. The prophage regions were predicted with PhiSpy 4.2.19, which uses typical prophage features (e.g. gene length, strand directionality, AT/GC skews, insertion points) [52].

### Phylogenetic and evolutionary analyses

The taxonomic position of *P. ananatis* Lstr was inferred using a multilocus sequence analysis (MLSA) that concatenated DNA sequences of five housekeeping genes (*fusA*, *gyrB*, *leuS*, *rpoB*, and *pyrG*) [53]. The MLSA approach has been found to provide robust and reliable results for species delineation in *Pantoea* [54]. A total of 48 *P *. *ananatis* contig level genomes and 11 genomes of their relatives including *P. agglomerans* and *E. amylovora* (Table S1, available in the online version of this article) were downloaded from the NCBI RefSeq assembly database to build phylogenetic trees [55, 56]. The sequences were aligned with MAFFT 7.407 [57], and were trimmed with trimAl v1.4.rev22 [58]. A maximum likelihood (ML) tree was constructed with IQ-TREE 1.6.5 [59] with a best-fitting nucleotide substitution model calculated with ModelFinder [60]. Node support was calculated with 1000 ultrafast bootstraps. In addition, we constructed a genome-wide species tree with concatenated protein sequences of single-copy orthologs that were determined by OrthoFinder 2.2.6 [61]. The phylogenetic trees were visualized with ggtree [62] and annotated in Inkscape (https://inkscape.org/).

The evolutionary rate of bacteria was estimated using the Bayesian Markov chain Monte Carlo method implemented in BEAST2 2.6 [63]. The strict molecular clock model was selected, and the optimal model and parameters of the nucleotide replacement model were obtained with the bModelTest software [64]. We considered the collection time of the bacteria was the time when the sequencing data were published. The highest probability density of 95 % (HPD) was shown as the range of evolutionary rates. All other priorities were set to the default values assigned by the software.

### Comparative genomic analyses

*P. ananatis* Lstr was further compared with 13 complete *P. ananatis* genomes (Table S2). Synteny between the genomes was analysed with NUCmer and visualized with mummerplot on Mummer 4.0.0beta2 [65] and the Dual Synteny Plot in TBtools (v1.0697) [66]. The average nucleotide identities (ANI) between *P. ananatis* genomes were calculated using OrthoANI 1.40 [67]. Ortholog analysis across multiple genomes was performed using OrthoFinder 2.2.6 [61] and DIAMOND v0.8.38.100 [68]. The number of orthogroups across *P. ananatis* was visualized as UpSet plots using the R package UpSetR 1.4.0 [69].

The *P. ananatis* genomes were annotated as described above and compared by different groups. Genomes of *P. agglomerans* and *E. amylovora* were also included in analysing bacterial genomic characters and COGs. The number of genes in each IS family and COG category for each genome was subjected to Principal Coordinates Analysis (PCoA) using the R package vegan 2.5–7 [70]. The genomic features were compared with the Wilcoxon Rank Sum test. All statistical analyses were performed with R [35].

### Identification of T6SS pathways

T6SS pathway genes were identified with ortholog analysis by OrthoFinder 2.2.6 [61], by searching similar proteins of known proteins with blastp 2.9.0+ [71] and by manually checking gene annotations. We followed Shalom’s [72] method to define the nomenclature of T6SS genes. The phylogeny of TssM and Hcp protein sequences was constructed with IQ-TREE as described above. The operon structures were visualized with the R package ‘genoPlotR’ [73].

## Results

### Localization of *Pantoea* in *L. striatellus*

**Fig. 1.**
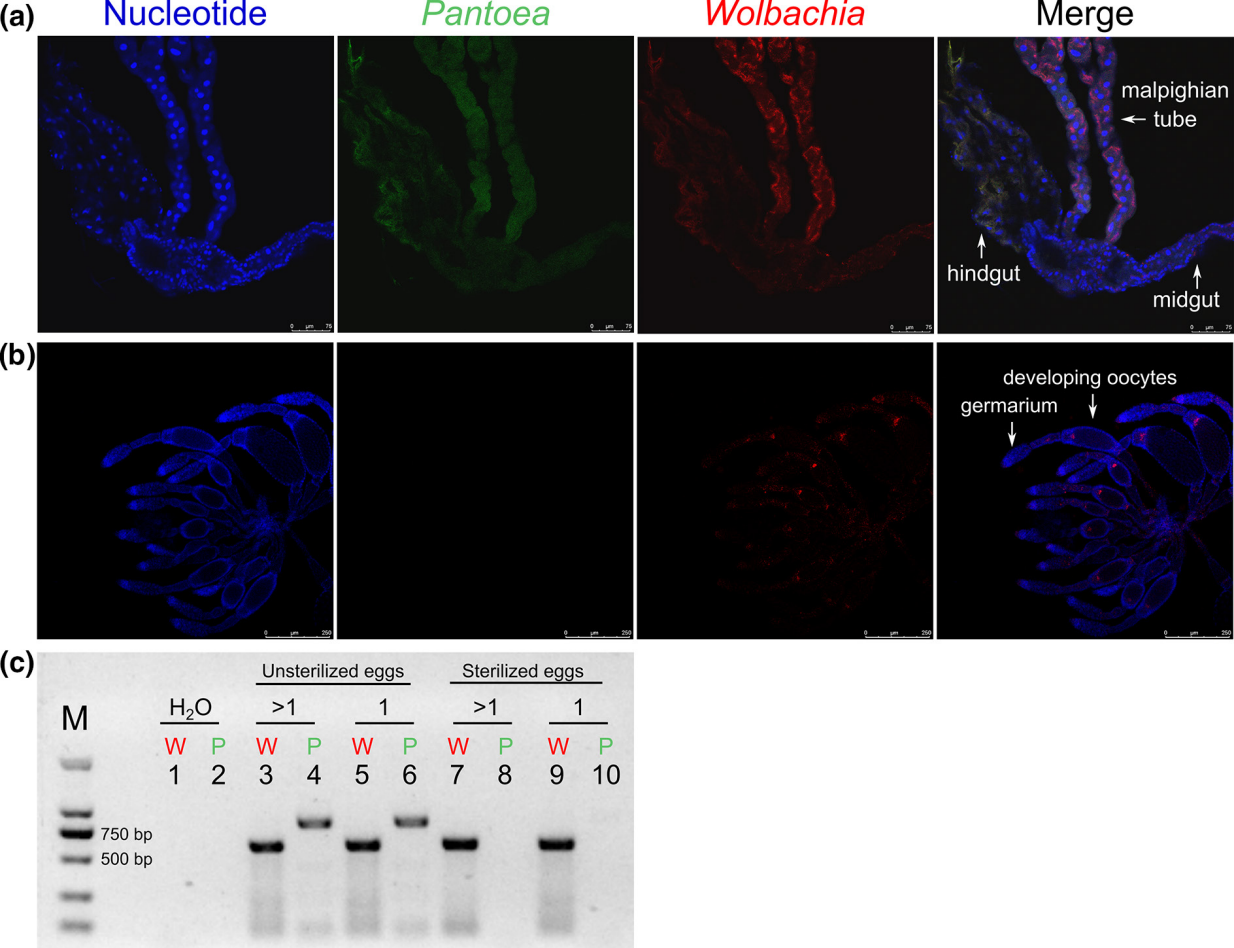
Localization of *Pantoea* and *Wolbachia* in *L. striatellus*. FISH of the midgut (**a**) and ovary (**b**). The tissues were labeled with the nuclear DNA stain (DAPI, blue), *Pantoea *-specific probe (FAM, green), and *Wolbachia *-specific probes (rhodamine, red). (**c**) PCR detection of *Pantoea* and *Wolbachia* in *L. striatellus* eggs. Lanes 1–2, negative controls; lanes 3–4, unsterilized multiple eggs; lanes 5–6, unsterilized single egg; lanes 7–8, sterilized multiple eggs; lanes 9–10, sterilized single egg. Lane odd numbers and W (red), amplification results using *Pantoea* primers; lane even numbers and P (green), amplification results using *Wolbachia* primers.

The FISH results revealed that *Pantoea* was present in the digestive system, including the midgut, hindgut, and Malpighian tubes (Fig. 1a), but not in the reproductive organ or oocytes (Fig. 1b). On the contrary, the signals of the intracellular symbiont *Wolbachia,* a reproductive manipulator that can be vertically transmitted among planthoppers [74], were observed in both guts and oocytes. We did not find any positive signals for either bacterium in the negative controls (Fig. S1). In addition, *Pantoea* was detected in unsterilized planthopper eggs with diagnostic PCR, but not in surface-sterilized eggs (Fig. 1c), indicating *Pantoea* cannot be vertically transmitted in *L. striatellus*.

### Pathogenicity of *P. ananatis* Lstr to *L. striatellus*

**Fig. 2.**
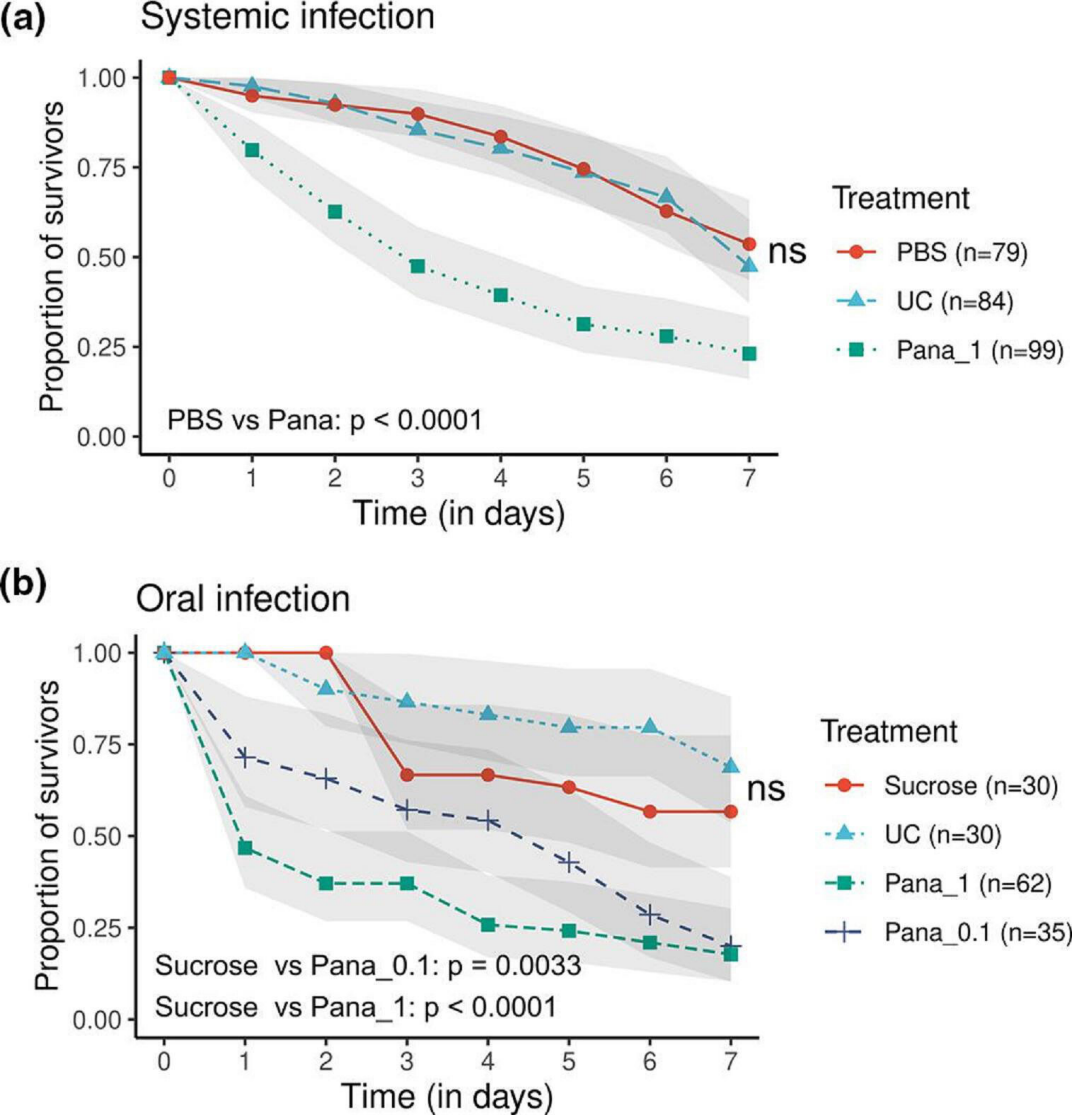
Survival of *L. striatellus* planthoppers after systemic and oral infection of *P. ananatis *. Survival curves represent the average percent survival and grey ribbons represent a 95 % confidence interval. The ‘ns’ symbol between survival curves of UC (unchallenged) and control treatments (PBS or sucrose) indicates no significant difference (*P* > 0.05). The *P*-values on each figure indicate the difference between bacterial infection and PBS prick or sucrose controls. Pana_0.1 and Pana_1 indicate infection of *P. ananatis* Lstr with OD_600_ = 0.1 and 1, respectively.

To isolate *P. ananatis,* we sequenced the 16S rRNAs of several bacterial colonies obtained from homogenized *L. striatellus*. We identified one strain and called it the Lstr strain. To evaluate its pathogenicity against rice planthoppers, we first measured the survival of planthoppers after septic injury (see Methods). As insects have a circulatory system, the septic injury infection by injecting microbes into insects can bypass natural barriers and causes systemic infection rapidly, making insects more susceptible. Systemic infection of *P. ananatis* (OD_600_=1) killed 77 % *L*. *striatellus* after 7 days of infection, significantly more than that of the PBS control (46.4 %, *P*<0.0001) (Fig. 2a). The mortalities of males and females in response to the pricking treatments were similar (Fig. S2). We next orally infected planthoppers by feeding them 2.5 % sucrose solutions containing *P. ananatis* and monitored their survival. *L. striatellus* adults treated with both concentrations of *P. ananatis* (OD_600_=0.1 and 1) experienced more than 80 % mortality at 7 days after bacterial infection (Fig. 2b), significantly higher than did the sucrose control (*P*<0.001 for both concentrations).

### Characteristics of *P. ananatis* Lstr genome

To gain evolutionary insights about *P. ananatis* Lstr, we sequenced its genome. The assembled *P. ananatis* Lstr genome contains one chromosome and one plasmid. The PGAP pipeline annotation results revealed the genome contained a total of 4644 genes, including 4521 protein-coding sequences (4394 CDSs and 127 pseudogenes). The total length of the genome is 4.95 Mb, which is within the typical range of *P. ananatis* genomes (4.34~5.28 Mb) (Table S2). The average GC is 52.48 %, the lowest value reported for *P. ananatis* genomes so far (Table S2).

### Taxonomy of *P. ananatis* Lstr

**Fig. 3.**
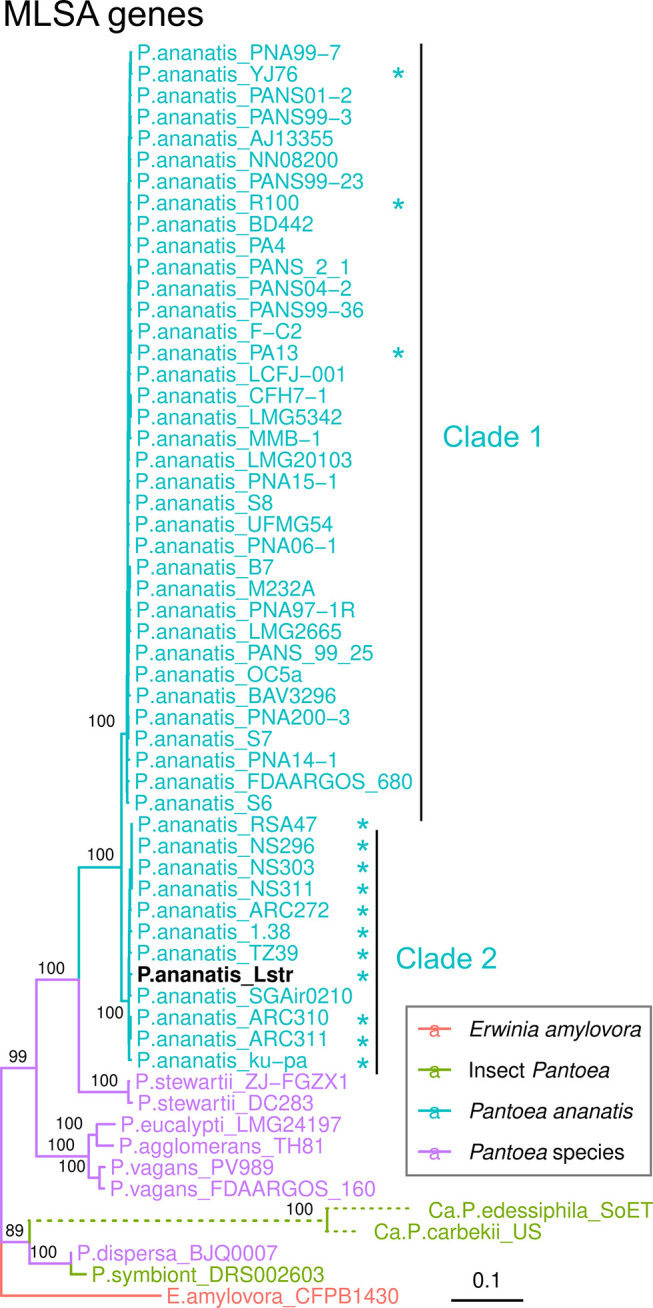
Phylogenetic relationship of *Pantoea* species. The maximum likelihood tree was calculated with a concatenated DNA sequence of five MLSA genes (*fusA, gyrB, leuS, rpoB*, and *pyrG*) sequences (12 751 bp) using a TNe+R10 model. The categories of bacteria are colour coded as shown on the branches. Because the branches of *Ca*. *Pantoea* were too long, they were shortened as dotted lines. The symbol * indicates that strain was isolated from rice-associated sample. Bootstrap values are indicated at the respective nodes. The scale bar represents the average number of substitutions per site. Accession numbers of sequences in this tree were listed in Table S1.

The taxonomic status of *P. ananatis* Lstr was confirmed by phylogenomic analyses using the five genes in the multilocus sequence analysis (MLSA) (*fusA*, *gyrB*, *leuS*, *rpoB*, and *pyrG*) and protein sequences of 319 concatenated single-copy orthologs that were obtained from *Pantoea* genomes, respectively (Fig. 3, Fig. S3, available in the online Supplementary Material). *P. ananatis* Lstr was in a cluster distinct from other known insect-associated *Pantoea* (such as *Candidatus Pantoea edessiphila* and *Ca*. *P. carbekii *) [75]. This *P. ananatis* cluster grouped into two mono-phylogenetic clades, clades one and two (Fig. 3). Clade two contains the Lstr strain and 11 other strains (Table S1). All but one of the members of clade two were isolated from rice-related samples, such as leaves or seeds of rice (*Oryza sativa*) and *Oryza sp*, collected from locations far distant away from each other, which includes South Asia, Eastern Asia, Europe and West Africa (Table S1). The exception was SGAir0210, which is a *P. ananatis* strain isolated from the air in Singapore. The finding of *P. ananatis* clade two strains in rice from many parts of the world suggests that it has evolved with rice over a long time.

### Characteristics of *P. ananatis* Lstr and other *Pantoea* genomes

**Fig. 4.**
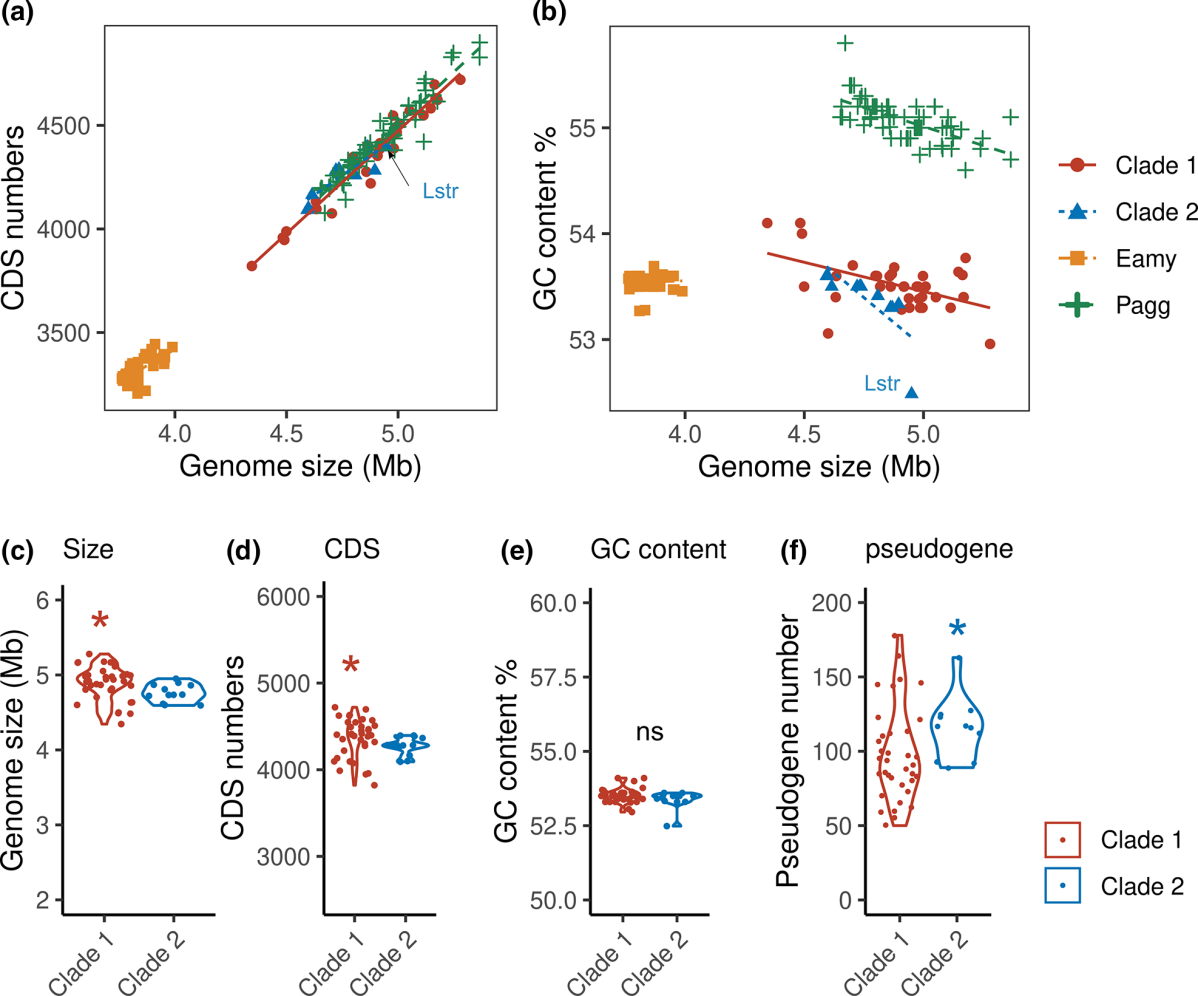
Comparison of genomic characteristics of *P. ananatis* clades. Comparison of genome size and CDS numbers (**a**) and %G+C content (**b**) of *E. amylovora* (Eamy)*, P. agglomerans* (Pagg) and *P. ananatis* (Clade 1 and Clade 2) bacteria. Comparison of genomes of *P. ananatis* bacteria on genome size (**c**), CDS numbers (**d**), %G+C content (**e**), and pseudogene numbers (**f**). ns, not significantly different; *, *P* < 0.05. A red star indicates higher mean of Clade 1 and a blue star means higher mean of Clade 2.

The genomes of *P. ananatis* and *P. agglomerans* were much larger than the genome of their sister genus *E. amylovora* (Fig. 4a). However, the GC contents of *P. ananatis* and *E. amylovora* were similar and much lower than the GC content of *P. agglomerans* (Fig. 4b). Within *P. ananatis,* the genomes of clades one and two have similar GC contents (mean clade one=53.5 %, mean clade two=53.4 %, Wilcoxon rank sum test, W=262, *P*=0.273), but clade two members have 1) significantly smaller genomes (clade one=4.89, clade two=4.75, Wilcoxon rank sum test, W=316, *P*=0.016), 2) much fewer protein coding sequences (CDSs) (clade one=4365, clade two=4266, Wilcoxon rank sum test, W=307, *P*=0.031) and 3) more pseudogenes (clade one=98, clade two=114, Wilcoxon rank sum test, W=127, *P*=0.035) (Fig. 4c – f). These results suggest that clade two has undergone a genome reduction.

The average nucleotide identity (ANI) between the genomes of *P. ananatis* Lstr and the other clade two members is 99.13 %, which is much higher than it is between *P. ananatis* Lstr and clade one members (Wilcoxon rank-sum test, *P*<0.001) (Table S3). The average of the ANIs between Lstr and clade one strains is 96.5 %, which is slightly higher than 95 %, the ANI value proposed as a recommended cut-off for species delineation [76]. As the directions of scaffolds of draft genomes are artificially rearranged during the MUMmer analysis, we compared the structural variations between *P. ananatis* Lstr and other ‘complete level’ *Pantoea* species from NCBI. The MUMmer dot plots and synteny figure show that macroscopic differences between *P. ananatis* Lstr and *P. ananatis* species were fewer than those compared with Lstr and other *Pantoea* species (Fig. S4). Together, these results indicate that the *P. ananatis* strains in clade one and clade two have different genomic structures.

### Mobile elements

Mobile elements, such as prophage sequences (which can produce phages if specifically activated) and insertion sequences (ISs) have key roles in driving horizontal gene transfer and microbial genome evolution [51, 77]. PhiSpy identified four prophage regions (pp1-4) in the *P. ananatis* Lstr genome, with a combined size of 115.9 kb (2.3 % of the total genome, Table S4). In contrast to the intracellular bacteria *Wolbachia* in *L. striatellus,* whose prophage regions contain genes of mostly IS elements and ankyrin repeat (ANK)-containing proteins, the *P. ananatis* Lstr prophage regions are mainly composed of phage structural proteins and enzymes (Table S5). For example, the genes on pp3 encode many Mu-like prophage structural proteins, such as the tail sheath protein gpL, DNA circulation protein, and four tail proteins. One of the prophages (pp4) encodes proteins of assembly and secretion of P pili that are required for adhesion of pathogenic bacteria [78]. The number of prophages in *P. ananatis* did not differ significantly between clades one and two (Wilcoxon rank-sum test, W=160, *P*=0.262).

**Fig. 5.**
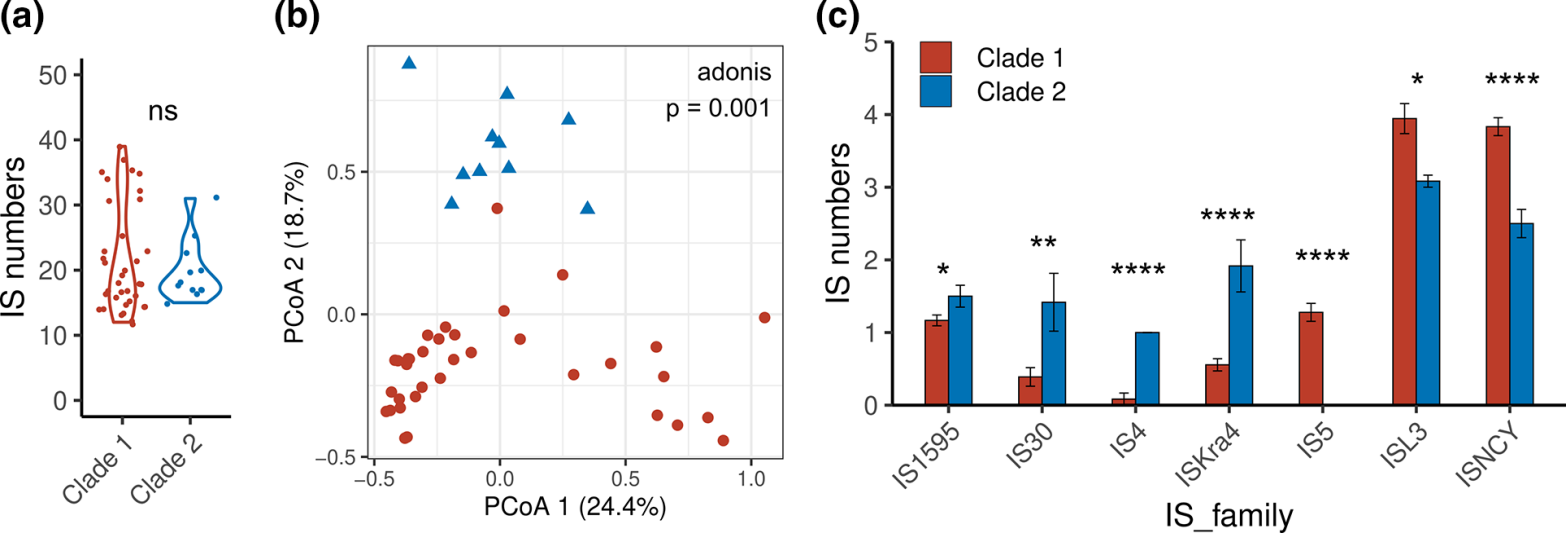
Comparison of IS elements from different *P. ananatis* clades. (**a**) Numbers of IS numbers. (**b**) PCoA analysis based on the annotated IS genes. (**c**) Bar chart comparing the gene numbers in each IS family between Clades 1 and 2. *P ≤ 0.05; **P ≤ 0.01; ***P ≤ 0.001; ****P ≤ 0.0001.

The ISfinder database identified 31 IS elements in nine IS families from the *P. ananatis* Lstr genome (Table S6), which is the highest number in clade two. The IS3 family, which is the most abundant IS family in *P. ananatis* Lstr, is also the most abundant IS family in *Wolbachia *, which is the most abundant intracellular symbiont in *L. striatellus* [77]. The total number of IS genes in the bacteria in clade one is similar to that in clade two (Wilcoxon rank-sum test, W=212.5, *P*=0.943) (Fig. 5a). However, the IS elements are significantly different between the two clades. The PCoA results show that the distributions of the IS genes in each family were significantly different between clades one and two (adonis test, R^2^=0.2, *P*=0.001) (Fig. 5b). The IS5 family was detected only in clade one (Fig. 5c). The clade two genomes harbour more IS1595, IS30, and IS4 family genes than do the clade one genomes.

### Functional categories of *P. ananatis* genes

**Fig. 6.**
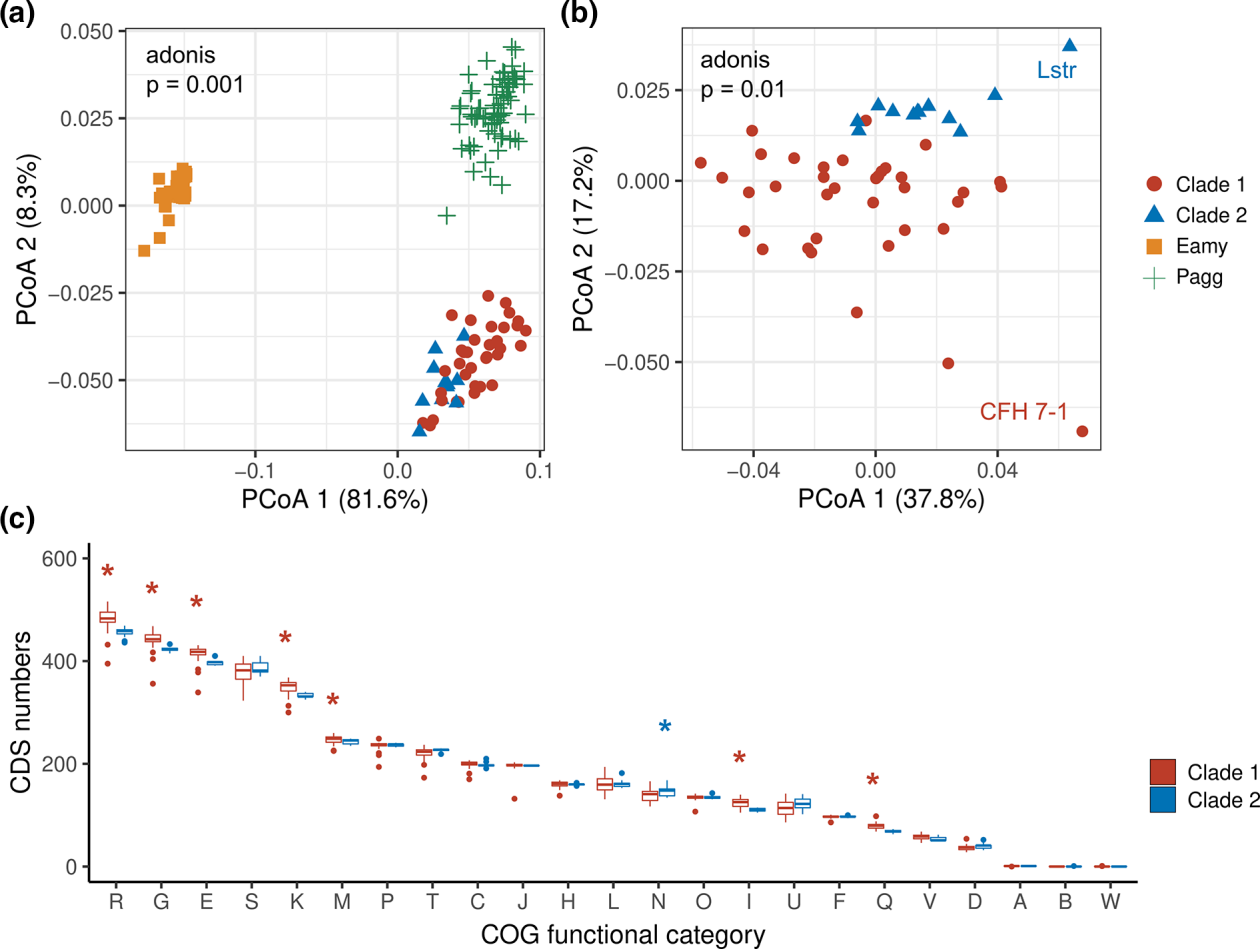
Comparison of COG categories from *P. ananatis *. (**a**) PCoA analysis of genomes of *E. amylovora* (Eamy)*, P. agglomerans* (Pagg) and *P. ananatis* (Clades 1 and 2). (**b**) PCoA analysis of only *P. ananatis* genomes. (**c**) Boxplots comparing COG categories between *P. ananatis* Clades 1 and 2. Abbreviations of COG categories were from the NCBI Database of Clusters of Orthologous Genes (https://www.ncbi.nlm.nih.gov/research/cog/). A: RNA processing and modification, B: Chromatin structure and dynamics, C: Energy production and conversion; D: Cell cycle control, cell division, chromosome partitioning; E: Amino acid transport and metabolism; F: Nucleotide transport and metabolism; G: Carbohydrate transport and metabolism; H: Coenzyme transport and metabolism; I: Lipid transport and metabolism; J: Translation, ribosomal structure, and biogenesis; K: Transcription; L: Replication, recombination and repair; M: Cell wall/membrane/envelope biogenesis; N: Cell motility; O: Posttranslational modification, protein turnover, chaperones; P: Inorganic ion transport and metabolism; Q: Secondary metabolites biosynthesis, transport and catabolism; R: General function prediction only; S: Function unknown; T: Signal transduction mechanisms; U: Intracellular trafficking, secretion, and vesicular transport; V: Defence mechanisms; W: Extracellular structures.

We next identified the functional categories of the CDSs by assigning them to COG and KEGG pathways. The PCoA figure based on the number of genes in each of the COG categories showed that *E. amylovora* and *Pantoea* spp. clustered separately by PCoA1 (81.6 % of total variance). In contrast, PC2 clearly separates *P. ananatis* and *P. agglomerans* by PCoA2 (8.3 % of total variance) (Fig. 6a). Similar to the analysis of IS elements, the PCoA analysis within *P. ananatis* confirmed the clear separation of the clade one and two genomes (adonis test, R^2^=0.09, *P*=0.01) (Fig. 6b). Both the CFH7-1 and Lstr strain of *P. ananatis* were at the far right in the PCoA plot (Fig. 6b), indicating they may share some similar characters. The CFH7-1 strain was reported to be transmitted by a Hemipteran cotton pest, *Pseudatomoscelis seriatus* [79]. Additionally, clade one *P *. *ananatis* encodes significantly more genes belonging to metabolism-related COG categories (i.e. G, E, I, Q, R, K, M) than do the clade two genomes (Fig. 3). Only genes related to cell motility (N) were more numerous in clade two than in clade one (*P*<0.05) (Fig. 3c). These findings on gene categories indicate that the two *P. ananatis* clades evolved differently.

We measured chromosome-wide changes in CDSs of *P. ananatis* clade one and clade two members over time to compare their estimated rates of molecular evolution. Our estimates for clades one and two overlap and range from 17.2 to 31 700 changes and 2.59 to 16 300 changes per ten billion positions per year, respectively (Fig. S5). Some genes had lower estimates in clade two than in clade one, which implies these genes evolved more slowly in clade two. Our estimate of the evolutionary rate of *P. ananatis* overlaps with rates calculated for some fast-evolving pathogens (e.g. *Enterococcus faecium* and *Acinetobacter baumannii *) [80], but was much smaller than the evolutionary rates observed in the poultry pathogen *Mycoplasma gallisepticum* and insect symbiont *Spiroplasma *. Indeed, the substitution rates in *P. ananatis* fall at around the lower estimates for DNA viruses and were much slower than those of RNA viruses (Fig. S5).

### Ortholog analysis

**Fig. 7.**
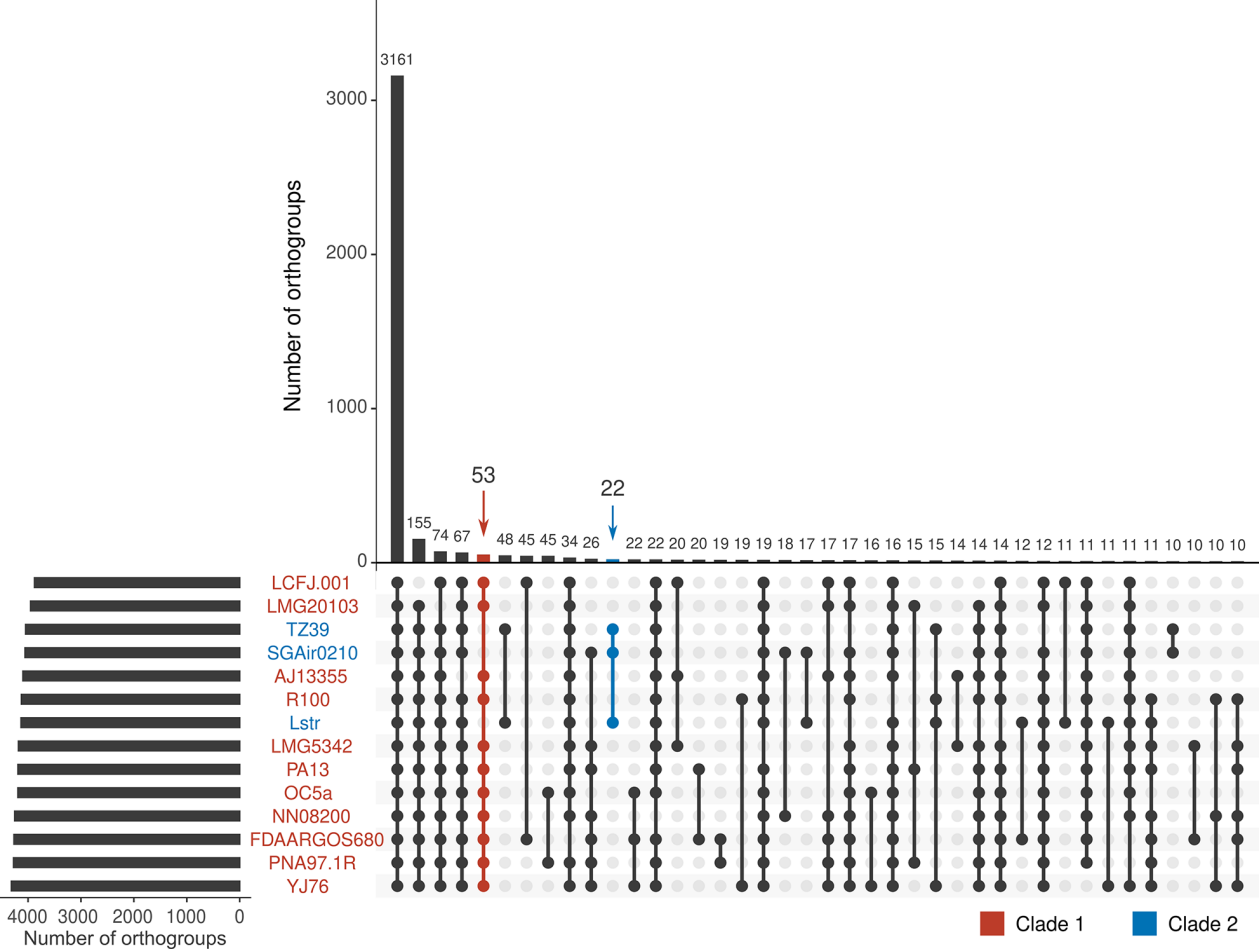
Orthology analysis of proteins from all complete *P. ananatis* genomes identifies core *P. ananatis* proteome. The set of all 60 850 proteins from Lstr and 12 other completed *P. ananatis* genomes were grouped into 5140 orthogroups using the OrthoFinder software. Of these, 3161 orthogroups were present across all the genomes, representing the core *P. ananatis* proteome, represented by the first bar in the Upset plot. Filled dots denote presence and empty dots indicate absence of orthogroups in each bacterium. Orthogroups present in all Clade 1 genomes but absent in all Clade 2 genomes were colored in red, while orthogroups of the opposite is in blue. The largest 40 intersections/overlaps between orthogroups are displayed.

Coding proteins from the 14 published complete level *P. ananatis* genomes were analysed using the OrthoFinder programme [61]. The core proteome, defined as the set of proteins present in all genomes analysed, consists of 3161 orthogroups (Fig. 7), and 3048 of these orthogroups were single-copy orthologs. COG cluster analysis showed that single-copy orthologs are mainly involved in basic biological processes (Table S7).

For *P. ananatis* Lstr, 112 proteins (2.5%) were Lstr-specific and were not assigned to any other orthogroups. The *P. ananatis* Lstr-specific proteins included transposase and phage-related proteins (11), and restriction endonucleases (two), both of which have key roles in horizontal gene transfer, and hypothetical proteins (76) (Table S7). In addition, we found 53 orthogroups that were present in all clade one genomes but absent in all clade two genomes, while 22 orthogroups were present in all clade two genomes and absent in all clade one genomes (Fig. 7). In particular, the clade two-specific proteins include MFS transporters (COG2814), LysR family, and TetR/AcrR family transcriptional regulators (COG0583), carbonic anhydrase (COG3338), nuclear transport factor two family protein (COG3631), ergot alkaloid biosynthesis protein (COG0702), and universal stress protein (COG0589) (Table S7).

### Type VI secretion system in *P. ananatis* Lstr

**Fig. 8.**
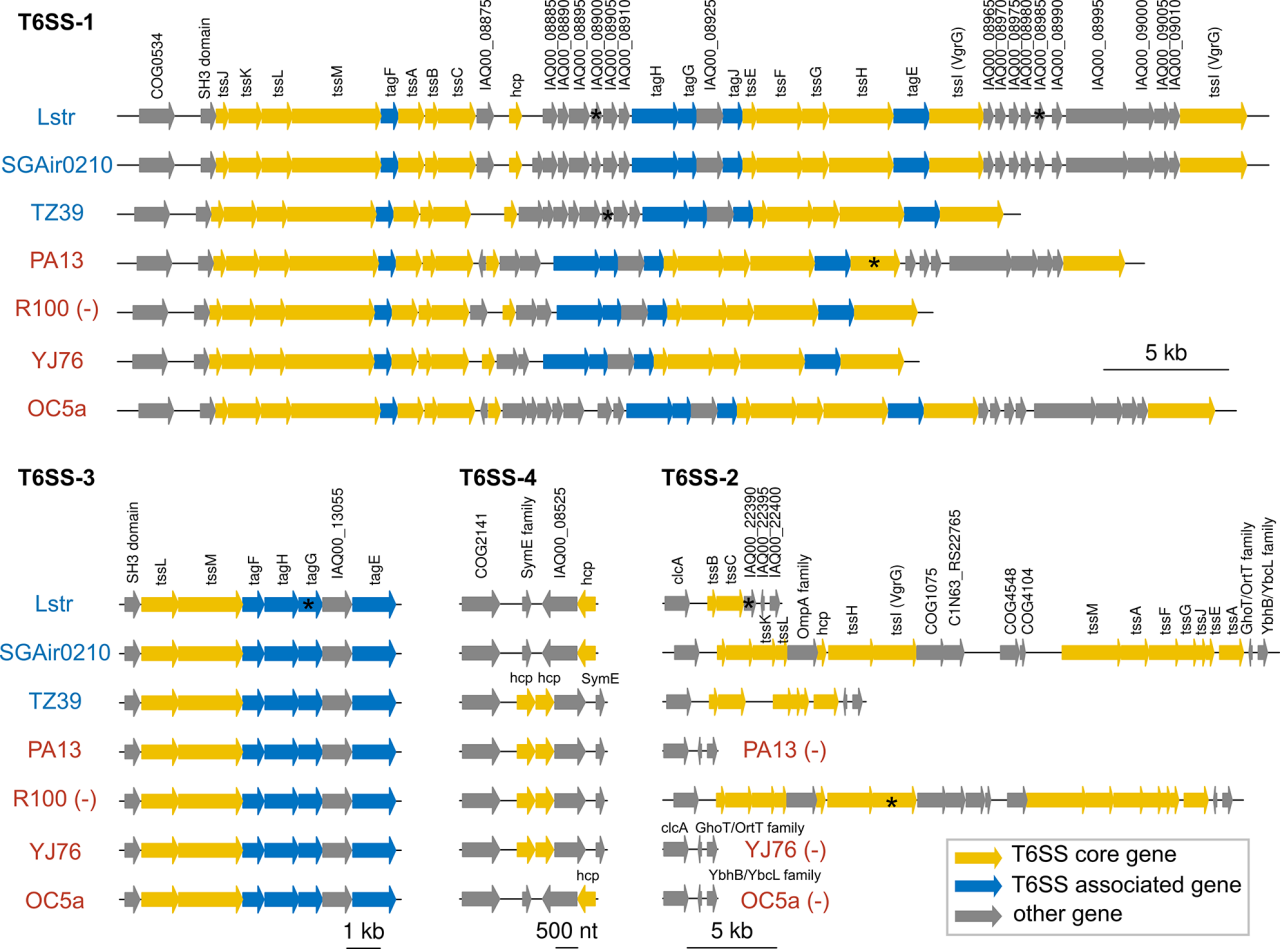
Genetic organization of the different T6SS gene clusters in *P. ananatis* genomes. Genes are indicated by arrows and the direction of the arrows represents the direction of transcription of the gene related to the rest of the genome. We used the type VI secretion system gene nomenclature of Shalom *et al*. [72]. The yellow arrows indicate T6SS core genes (*tssA-M*) while the blue arrows indicate T6SS associated genes (*tagA-P*). The other genes were colored in grey. The symbol * indicates a pseudogene. The symbol (-) indicates that the direction of operon was reversed. Clade 1 *P *. *ananatis* are colored in red font and the Clade 2 are blue.

*P. ananatis* Lstr has multiple secretory pathways including the Types I, II, V, and VI secretion systems (T1SS, T2SS, T5SS and T6SS, respectively), and the Sec, and Tat secretion systems (Table S5). The T3SS was missing in *P. ananatis* Lstr (Table S5). T6SS has been reported to be required for *P. ananatis *’s pathogenesis and bacterial competition [81]. The Lstr genome has a T6SS with 30 genes organized in four clusters and two individual genes (Fig. 8). The T6SS-1 cluster contains 14 core genes (*tssA-M*) and five associated genes (*tagE-H, TagJ*). As previously described [82], T6SS-1 and T6SS-3 were conserved among seven *P. ananatis* strains that were analysed. T6SS has variable regions that are different between the two clades. The Lstr, SGAir0210, and TZ39 (clade two) harbour more genes in the variable region one than do PA13, R100, and YJ76, which were isolated from rice as well but belong to clade one. OC5a, a clade one strain isolated from onion, encodes the same number of genes in variable region one as do clade two bacteria (Fig. 8). The T6SS-1s of Lstr, SGAir0210, PA13, and OC5a have an additional copy of *tssI/vgrG* effector compared to those of TZ39, R100, and YJ76.

The T6SS-2 cluster is present in the Lstr and R100 strains but completely missing in the PA13, YJ76, and OC5a strains. Moreover, the T6SS-2s of the Lstr, SGAir0210, and TZ39 strains have two, 14, and six T6SS genes, respectively, indicating that the T6SS-2 cluster in *P. ananatis* is diverse. In addition, we found that the T6SS-4 of all the analysed strains have genes encoding hemolysin co-regulated protein (Hcp) (Fig. 8). Phylogenetic analysis showed TssM was conserved among *Pantoea* species, but that of Hcp showed no convergence with *Pantoea *’s phylogeny (Fig. S6). Overall, the above results show that the numbers of many T6SS genes are highly variable and that these genes may have been involved in multiple horizontal gene transfers.

## Discussion

Our results show that the *P. ananatis* strain Lstr inhabits the digestive system of *L. striatellus* and is pathogenic to the host insect. The localization pattern of *P. ananatis* is similar to that found in onion thrips (*Thrips tabaci*) [15]. Other insect-associated *Pantoea* species, such as *P. agglomerans* and *Ca*. *Pantoea carbekii *, locate in the insect gut region as well [83, 84]. Unlike the intracellular symbiont *Wolbachia *, *P. ananatis* cannot enter the reproductive tissues of *L. striatellus*. The detection of *P. ananatis* DNA in unsterilized planthopper eggs but not in surface-unsterilized eggs is consistent with the results of *Ca. Pantoea carbekii*, which was abundant in the midgut and on the egg surface of stink bug (*Halyomorpha halys*) but was removed by bleach treatment [84]. Therefore, *P. ananatis* Lstr is not transmitted vertically among planthoppers. In thrips, *P. ananatis* can be horizontally transmitted through faeces but not through salivary secretions [15]. A recent study reports that the western corn rootworm also transmits *P. ananatis* to maize plants [13]. Together, these results suggest that *P. ananatis* Lstr may be transmitted horizontally.

*P. ananatis* Lstr naturally infects planthoppers and does not show obvious adverse phenotypes. Both systemic and gut infections of high dose *P. ananatis* caused significantly more mortality than the controls in our study, demonstrating that *P. ananatis* Lstr is mildly pathogenic to *L. striatellus*. However, *P. ananatis* had no significant effects on fitness parameters such as fecundity and development of onion thrips [16]. The *P. ananatis* strains from mulberry pyralid considerably reduced the cold hardiness of mulberry pyralid larvae by providing ice nucleation activities [9]. In stink bugs, the primary symbiont *Ca*. *Pantoea carbekii* contributes to host development by supplementing essential nutrients [84]. The mechanism of *P. ananatis* Lstr’s mild pathogenicity is unknown.

Although it was originally isolated from insects, *P. ananatis* Lstr is phylogenetically distinct from known insect-associated symbionts, such as *Ca*. *Pantoea carbekii *, *Ca*. *Pantoea edessiphia*, and *Ca*. *Pantoea persica*. Moreover, Lstr was positioned within the group of *P. ananatis* that we designated as clade two. Of the 12 analysed genomes in clade two, ten strains were isolated from the leaves or seeds of rice and *Oryza* sp., one strain (Lstr) was isolated from rice planthoppers, which feed on rice leaves, and the last one (SGAir0210 strain) was collected from the air. The places in which clade two bacteria were isolated are South Asia, Eastern Asia, Europe, and West Africa, which are well separated from each other. This suggests that these strains have been closely associated with rice plants for a long time.

Many genomes of recently evolved symbionts and pathogens contain many pseudogenes, many mobile elements, deletions, and chromosome rearrangements, which usually occur at the initial stage of genome reduction [85]. Therefore, compared to free-living bacteria, symbiotic bacteria usually have many characteristics of genome reduction, such as smaller genomes, fewer CDSs and smaller GC contents. We found that, compared to clade one, clade two *P *. *ananatis* have significantly smaller genomes and encode much fewer CDS but more pseudogenes, suggesting that clade two is at the early stage of genome erosion. In addition, Lstr showed a GC content of 52.48%, the smallest number in reported *P. ananatis* genomes, which suggests that Lstr has a co-evolutionary relationship with *L. striatellus*.

The numbers of prophages and ISs are similar between *P. ananatis* clades one and two. The prophages and ISs are mobile elements and contribute to the evolution of bacteria by mediating the horizontal transfer of clusters of genes. In addition, IS fragments tend to be deleted with adjacent DNA sequences and lead to a reduction in genome size [86]. In the bacterial pathogen *Aeromonas salmonicida *, ISs were shown to be involved in rearrangement events that cause the deletion of secretion system genes and lead to a loss of virulence [87]. The IS elements are significantly different between clades one and two, which supports the idea that the two clades underwent different evolutionary paths. Bacteriophages have been utilized to suppress bacterial palea browning of rice caused by *P. ananatis* [88]. The *P. ananatis* Lstr prophage regions encode enzymes such as a DNA modification methylase, a muraminidase, and periplasmic serine proteases that may facilitate their invasion of bacteria. P pili, which are required for the adhesion of pathogenic bacteria [78], were predicted to be synthesized by pp4 prophages (Table S5). Further studies are needed to determine what roles, if any, phage genes play in *P. ananatis* Lstr’s pathogenicity to *L. striatellus*.

Some *Pantoea* species require T3SSs for pathogenicity when interacting with hosts [50, 89]. The PSI-2 T3SS was identified in *P. ananatis* strains 15 320 and DAR76143 [50]. However, *P. ananatis* Lstr lacks genes homologous to T3SS but contains a complete T6SS. T6SSs are present in several human and plant pathogens. T6SSs have central roles in bacterial competition, pathogenesis, and host interactions [90]. *P. ananatis* harbours three T6SS loci, namely T6SS-1,–2, and −3 [82]. T6SS-1 and −3 are conserved among all *P. ananatis* strains [82]. The T6SS-1s of the PA13 and OC5a strains encode one more valine-glycine repeat (VgrG) effector protein than do the T6SS-1s of the TZ39, R100, and YJ76 strains, although all of these strains were isolated from rice. T6SS-2 is present in the clade two members but the number of T6SS genes vary widely among *P. ananatis* strains. Of the analysed clade one members, T6SS-2 is present in R100 but is missing in PA13, YJ76 and OC5a. In a phylogenetic analysis of concatenated TssB and TssC amino acid sequences, T6SS-2 and T6SS-1 genes were clustered in distinct groups and the T6SS-2 of *P. ananatis* was close to the T6SS-2s of *Erwinia* spp. and *Serratia* spp. [82], indicating that T6SS-2 was present in an ancient ancestor of *P. ananatis *. But why it is missing in many *P. ananatis* strains is unclear.

The T6SS-4s of *P. ananatis* harbour one or two duplicate copies of *hcp* and are first characterized in this study. The *hcp* of T6SS-4 is located near an LLM class flavin-dependent oxidoreductase (COG2141) and a type I addiction module toxin of the *SymE* family (IAQ00_08520 in Lstr). As the phylogeny of the Hcp proteins showed no convergence with *Pantoea *’s phylogeny, we hypothesize that T6SS-4 has experienced multiple horizontal gene transfers.

Shyntum *et al*. [81] deleted the conserved T6SS-1 and −3 gene clusters of *Pantoea ananatis* strain LMG 2665, respectively, and found that T6SS-1 plays an essential role in the pathogenicity of *P. ananatis* in onion plants, while deletion of T6SS-3 did not affect pathogenicity. They also found that T6SS-1 but not T6SS-3 was required to inhibit competitor bacteria. Additionally, that phenotypes of inhibiting competitors rely on *TssA* and *Hcp*, thus indicating that the T6SS-1 gene cluster encodes a functioning T6SS. The genes of T6SS-3 can be found in T6SS-1, but many T6SS-1 genes (such as *TssA* and *Hcp*) are found only in T6SS-1 in LMG 2665. It would be interesting to study whether T6SS-3 has functions that complement the functions of T6SS-1. For example, do deletions of *TssL* and *TssM* in T6SS-1 change the pathogenicity of *P. ananatis *?

In conclusion, our results show that the *P. ananatis* Lstr behaves more like an insect gut symbiont but is also pathogenic to the host. Our analysis of the *P. ananatis* Lstr genome and related genomes show that *P. ananatis* can be grouped into two clades (clades one and two) and that Lstr is in clade two. In particular, the strains of clade two contain mainly bacteria isolated from rice or rice-associated samples. The *P. ananatis* clade two members have significantly smaller genomes, and much fewer CDS but more pseudogenes, which suggests that they may be at the early stage of genome reduction [85]. Though the observed variations of T6SS were not distinguished between the two clades, further studies are needed to clarify whether the genome reduction of clade two *P *. *ananatis* strains is related to their close association with rice. This study has clarified our understanding of the phylogenetic diversity of *P. ananatis* and provides further clues to the interactions between *P. ananatis* and rice that may lead to advances in rice protection and pest control.

## Supplementary Data

Supplementary material 1

Supplementary material 2

Supplementary material 3

Supplementary material 4

Supplementary material 5

Supplementary material 6

Supplementary material 7

Supplementary material 8

Supplementary material 9

## References

[1] Weller-Stuart T, De Maayer P, Coutinho T (2017). *Pantoea ananatis*: genomic insights into a versatile pathogen. Mol Plant Pathol.

[2] Serrano FB (1928). Bacterial fruitlet brown-rot of pineapple in the philippines. Philipp J Sci.

[3] Yu L, Yang C, Ji Z, Zeng Y, Liang Y (2022). First report of new bacterial leaf blight of rice caused by *Pantoea ananatis* in Southeast China. Plant Dis.

[4] Gitaitis R, Walcott R, Culpepper S, Sanders H, Zolobowska L (2002). Recovery of *Pantoea ananatis*, causal agent of center rot of onion, from weeds and crops in Georgia, USA. Crop Protection.

[5] Coutinho TA, Preisig O, Mergaert J, Cnockaert MC, Riedel K-H (2002). Bacterial blight and dieback of *Eucalyptus* species, hybrids, and clones in South Africa. Plant Dis.

[6] Coutinho TA, Venter SN (2009). *Pantoea ananatis*: an unconventional plant pathogen. Mol Plant Pathol.

[7] Kim HJ, Lee JH, Kang BR, Rong X, McSpadden Gardener BB (2012). Draft genome sequence of *Pantoea ananatis* B1-9, a nonpathogenic plant growth-promoting bacterium. J Bacteriol.

[8] Takahashi K, Watanabe K, Sato M (1995). Survival and characteristics of ice nucleation-active bacteria on mulberry trees (*Morus* spp.) and in mulberry pyralid (*Glyphodes pyloalis*). Jpn J Phytopathol.

[9] Watanabe K, Sato M (1999). Gut colonization by an ice nucleation active bacterium, *Erwinia* (*Pantoea*) *ananas* reduces the cold hardiness of mulberry pyralid larvae. Cryobiology.

[10] Watanabe K, Kawakita H, Sato M (1996). *Epiphytic bacterium*, *Erwinia ananas*, commonly isolated from rice plants and brown planthoppers (*Nilaparvata lugens*) in hopperburn patches. Appl entomol Zool.

[11] Bell AA, Medrano EG, López JD, Luff R Transmission and importance of *Pantoea ananatis* during feeding on cotton buds (*Gossypium hirsutum L*.) by cotton fleahoppers (*Pseudatomoscelis seriatus reuter*). world cotton research conference-42007.

[12] Gitaitis RD, Walcott RR, Wells ML, Perez JCD, Sanders FH (2003). Transmission of *Pantoea ananatis*, causal agent of center rot of onion, by tobacco thrips, *Frankliniella fusca*. Plant Dis.

[13] Krawczyk K, Foryś J, Nakonieczny M, Tarnawska M, Bereś PK (2021). Transmission of *Pantoea ananatis*, the causal agent of leaf spot disease of maize (*Zea mays*), by western corn rootworm (*Diabrotica virgifera virgifera* LeConte). Crop Protection.

[14] Acevedo FE, Peiffer M, Tan C-W, Stanley BA, Stanley A (2017). Fall armyworm-associated gut bacteria modulate plant defense responses. Mol Plant Microbe Interact.

[15] Dutta B, Barman AK, Srinivasan R, Avci U, Ullman DE (2014). Transmission of *Pantoea ananatis* and *P. agglomerans*, causal agents of center rot of onion (*Allium cepa*), by onion thrips (*Thrips tabaci*) through feces. Phytopathology.

[16] Dutta B, Gitaitis R, Barman A, Avci U, Marasigan K (2016). Interactions between *Frankliniella fusca* and *Pantoea ananatis* in the center rot epidemic of onion (*Allium cepa*). Phytopathology.

[17] Yan H, Yu SH, Xie GL, Fang W, Su T (2010). Grain discoloration of rice caused by *Pantoea ananatis* (synonym *Erwinia uredovora*) in China. Plant Dis.

[18] Lu L, Chang M, Han X, Wang Q, Wang J (2021). Beneficial effects of endophytic *Pantoea ananatis* with ability to promote rice growth under saline stress. J Appl Microbiol.

[19] Xue Y, Hu M, Chen S, Hu A, Li S (2021). *Enterobacter asburiae* and *Pantoea ananatis* causing rice bacterial blight in China. Plant Dis.

[20] Mondal KK, Mani C, Singh J, Kim JG, Mudgett MB (2011). A new leaf blight of rice caused by *Pantoea ananatis* in India. Plant Dis.

[21] Wu L, Liu R, Niu Y, Lin H, Ye W (2016). Whole genome sequence of *Pantoea ananatis* R100, an antagonistic bacterium isolated from rice seed. J Biotechnol.

[22] Kini K, Lefeuvre P, Poulin L, Silué D, Koebnik R (2020). Genome resources of three West African strains of *Pantoea ananatis* causing bacterial blight and grain discoloration of rice. Phytopathology.

[23] Ma J, Zhang K, Huang M, Hector SB, Liu B (2016). Involvement of Fenton chemistry in rice straw degradation by the lignocellulolytic bacterium *Pantoea ananatis* Sd-1. Biotechnol Biofuels.

[24] Nault LR, Denno RF, Perfect TJ (1994). Planthoppers: Their Ecology and Management.

[25] Bing XL, Zhao DS, Peng CW, Huang HJ, Hong XY (2020). Similarities and spatial variations of bacterial and fungal communities in field rice planthopper (*Hemiptera*: *Delphacidae*) populations. Insect Sci.

[26] De Maayer P, Chan WY, Rubagotti E, Venter SN, Toth IK (2014). Analysis of the *Pantoea ananatis* pan-genome reveals factors underlying its ability to colonize and interact with plant, insect and vertebrate hosts. BMC Genomics.

[27] Sheibani-Tezerji R, Naveed M, Jehl M-A, Sessitsch A, Rattei T (2015). The genomes of closely related *Pantoea ananatis* maize seed endophytes having different effects on the host plant differ in secretion system genes and mobile genetic elements. Front Microbiol.

[28] Stice SP, Stumpf SD, Gitaitis RD, Kvitko BH, Dutta B (2018). *Pantoea ananatis* genetic diversity analysis reveals limited genomic diversity as well as accessory genes correlated with onion pathogenicity. Front Microbiol.

[29] Bing X-L, Winkler J, Gerlach J, Loeb G, Buchon N (2021). Identification of natural pathogens from wild *Drosophila suzukii*. Pest Manag Sci.

[30] Li TP, Zhou CY, Zha SS, Gong JT, Xi Z (2020). Stable establishment of *Cardinium* spp. in the brown planthopper *Nilaparvata lugens* despite decreased host fitness. Appl Environ Microbiol.

[31] Cole JR, Wang Q, Cardenas E, Fish J, Chai B (2009). The ribosomal database project: improved alignments and new tools for rRNA analysis. Nucleic Acids Res.

[32] Zhao DX, Zhang XF, Chen DS, Zhang YK, Hong XY (2013). *Wolbachia*-host interactions: host mating patterns affect *Wolbachia* density dynamics. PLoS ONE.

[33] Bing X-L, Xia W-Q, Gui J-D, Yan G-H, Wang X-W (2014). Diversity and evolution of the *Wolbachia* endosymbionts of *Bemisia* (*Hemiptera: Aleyrodidae*) whiteflies. Ecol Evol.

[34] Braig HR, Zhou W, Dobson SL, O’Neill SL (1998). Cloning and characterization of a gene encoding the major surface protein of the bacterial endosymbiont *Wolbachia pipientis*. J Bacteriol.

[35] R Team C (2022). R: a language and environment for statistical computing. http://www.R-project.org/.

[36] Chin C-S, Alexander DH, Marks P, Klammer AA, Drake J (2013). Nonhybrid, finished microbial genome assemblies from long-read SMRT sequencing data. Nat Methods.

[37] Myers EW, Sutton GG, Delcher AL, Dew IM, Fasulo DP (2000). A whole-genome assembly of *Drosophila*. Science.

[38] McKenna A, Hanna M, Banks E, Sivachenko A, Cibulskis K (2010). The genome analysis toolkit: a mapreduce framework for analyzing next-generation DNA sequencing data. Genome Res.

[39] Li R, Yu C, Li Y, Lam T-W, Yiu S-M (2009). SOAP2: an improved ultrafast tool for short read alignment. Bioinformatics.

[40] Li R, Li Y, Fang X, Yang H, Wang J (2009). SNP detection for massively parallel whole-genome resequencing. Genome Res.

[41] Li R, Li Y, Kristiansen K, Wang J (2008). SOAP: short oligonucleotide alignment program. Bioinformatics.

[42] Tatusova T, DiCuccio M, Badretdin A, Chetvernin V, Nawrocki EP (2016). NCBI prokaryotic genome annotation pipeline. Nucleic Acids Res.

[43] Shen W, Le S, Li Y, Hu F (2016). SeqKit: a cross-platform and ultrafast toolkit for FASTA/Q file manipulation. PLoS ONE.

[44] Cantalapiedra CP, Hernández-Plaza A, Letunic I, Bork P, Huerta-Cepas J (2021). eggNOG-mapper v2: functional annotation, orthology assignments, and domain prediction at the metagenomic scale. Mol Biol Evol.

[45] Kanehisa M, Sato Y, Morishima K (2016). BlastKOALA and GhostKOALA: KEGG tools for functional characterization of genome and metagenome sequences. J Mol Biol.

[46] Leimbach A (2016). Bac-genomics-scripts: bovine *E. coli* mastitis comparative genomics edition. Zenodo.

[47] Petersen TN, Brunak S, von Heijne G, Nielsen H (2011). SignalP 4.0: discriminating signal peptides from transmembrane regions. Nat Methods.

[48] Krogh A, Larsson B, von Heijne G, Sonnhammer EL (2001). Predicting transmembrane protein topology with a hidden Markov model: application to complete genomes. J Mol Biol.

[49] Eichinger V, Nussbaumer T, Platzer A, Jehl M-A, Arnold R (2016). EffectiveDB--updates and novel features for a better annotation of bacterial secreted proteins and Type III, IV, VI secretion systems. Nucleic Acids Res.

[50] Kirzinger MWB, Butz CJ, Stavrinides J (2015). Inheritance of *Pantoea* type III secretion systems through both vertical and horizontal transfer. Mol Genet Genomics.

[51] Siguier P, Perochon J, Lestrade L, Mahillon J, Chandler M (2006). ISfinder: the reference centre for bacterial insertion sequences. Nucleic Acids Res.

[52] Akhter S, Aziz RK, Edwards RA (2012). PhiSpy: a novel algorithm for finding prophages in bacterial genomes that combines similarity- and composition-based strategies. Nucleic Acids Res.

[53] Tambong JT (2019). Taxogenomics and Systematics of the Genus Pantoea. Front Microbiol.

[54] Tambong JT, Xu R, Kaneza CA, Nshogozabahizi JC (2014). An in-depth analysis of a multilocus phylogeny identifies leuS as a reliable phylogenetic marker for the genus *Pantoea*. Evol Bioinform Online.

[55] Haft DH, DiCuccio M, Badretdin A, Brover V, Chetvernin V (2018). RefSeq: an update on prokaryotic genome annotation and curation. Nucleic Acids Res.

[56] Benson DA, Cavanaugh M, Clark K, Karsch-Mizrachi I, Ostell J (2018). GenBank. Nucleic Acids Res.

[57] Katoh K, Standley DM (2013). MAFFT multiple sequence alignment software version 7: improvements in performance and usability. Mol Biol Evol.

[58] Capella-Gutiérrez S, Silla-Martínez JM, Gabaldón T (2009). trimAl: a tool for automated alignment trimming in large-scale phylogenetic analyses. Bioinformatics.

[59] Nguyen L-T, Schmidt HA, von Haeseler A, Minh BQ (2015). IQ-TREE: a fast and effective stochastic algorithm for estimating maximum-likelihood phylogenies. Mol Biol Evol.

[60] Kalyaanamoorthy S, Minh BQ, Wong TKF, von Haeseler A, Jermiin LS (2017). ModelFinder: fast model selection for accurate phylogenetic estimates. Nat Methods.

[61] Emms DM, Kelly S (2015). OrthoFinder: solving fundamental biases in whole genome comparisons dramatically improves orthogroup inference accuracy. Genome Biol.

[62] Yu G, Smith DK, Zhu H, Guan Y, Lam TY (2017). Ggtree: an r package for visualization and annotation of phylogenetic trees with their covariates and other associated data. Methods Ecol Evol.

[63] Bouckaert R, Heled J, Kühnert D, Vaughan T, Wu CH (2014). BEAST 2: a software platform for Bayesian evolutionary analysis. PLoS Comput Biol.

[64] Bouckaert RR, Drummond AJ (2017). bModelTest: Bayesian phylogenetic site model averaging and model comparison. BMC Evol Biol.

[65] Kurtz S, Phillippy A, Delcher AL, Smoot M, Shumway M (2004). Versatile and open software for comparing large genomes. Genome Biol.

[66] Chen C, Chen H, Zhang Y, Thomas HR, Frank MH (2020). TBtools: an integrative toolkit developed for interactive analyses of big biological data. Mol Plant.

[67] Yoon S-H, Ha S-M, Lim J, Kwon S, Chun J (2017). A large-scale evaluation of algorithms to calculate average nucleotide identity. Antonie Van Leeuwenhoek.

[68] Buchfink B, Xie C, Huson DH (2015). Fast and sensitive protein alignment using DIAMOND. Nat Methods.

[69] Conway JR, Lex A, Gehlenborg N (2017). UpSetR: an R package for the visualization of intersecting sets and their properties. Bioinformatics.

[70] Simpson GL, Solymos P, Stevens M, Wagner H (2010). Vegan: community ecology package. Time International.

[71] Camacho C, Coulouris G, Avagyan V, Ma N, Papadopoulos J (2009). BLAST+: architecture and applications. BMC Bioinformatics.

[72] Shalom G, Shaw JG, Thomas MS (2007). In vivo expression technology identifies a type VI secretion system locus in *Burkholderia pseudomallei* that is induced upon invasion of macrophages. Microbiol.

[73] Guy L, Kultima JR, Andersson SGE (2010). genoPlotR: comparative gene and genome visualization in R. Bioinformatics.

[74] Bing X-L, Zhao D-S, Hong X-Y (2019). Bacterial reproductive manipulators in rice planthoppers. Arch Insect Biochem Physiol.

[75] Otero-Bravo A, Goffredi S, Sabree ZL (2018). Cladogenesis and genomic streamlining in extracellular endosymbionts of tropical stink bugs. Genome Biol Evol.

[76] Kim M, Oh H-S, Park S-C, Chun J (2014). Towards a taxonomic coherence between average nucleotide identity and 16S rRNA gene sequence similarity for species demarcation of prokaryotes. Int J Syst Evol Microbiol.

[77] Bing XL, Zhao DS, Sun JT, Zhang KJ, Hong XY (2020). Genomic analysis of *Wolbachia* from *Laodelphax striatellus* (Delphacidae, Hemiptera) reveals insights into its “Jekyll and Hyde” mode of infection pattern. Genome Biol Evol.

[78] Mu X-Q, Bullitt E (2006). Structure and assembly of P-pili: a protruding hinge region used for assembly of a bacterial adhesion filament. Proc Natl Acad Sci.

[79] Medrano EG, Bell AA (2015). Genome sequence of *Pantoea ananatis* strain CFH 7-1, which is associated with a vector-borne cotton fruit disease. Genome Announc.

[80] Duchêne S, Holt KE, Weill F-X, Le Hello S, Hawkey J (2016). Genome-scale rates of evolutionary change in bacteria. Microb Genom.

[81] Shyntum DY, Theron J, Venter SN, Moleleki LN, Toth IK (2015). *Pantoea ananatis* utilizes a type VI secretion system for pathogenesis and bacterial competition. Mol Plant Microbe Interact.

[82] Shyntum DY, Venter SN, Moleleki LN, Toth I, Coutinho TA (2014). Comparative genomics of type VI secretion systems in strains of *Pantoea ananatis* from different environments. BMC Genomics.

[83] Zhang J, Huang Y, Huang X, Jiang M (2016). Infection state of *Pantoea agglomerans* in the rice water weevil *Lissorhoptrus oryzophilus* (Coleoptera: Curculionidae). Appl Entomol Zool.

[84] Kenyon LJ, Meulia T, Sabree ZL (2015). Habitat visualization and genomic analysis of “*Candidatus* Pantoea carbekii,” the primary symbiont of the brown marmorated stink bug. Genome Biol Evol.

[85] McCutcheon JP, Moran NA (2011). Extreme genome reduction in symbiotic bacteria. Nat Rev Microbiol.

[86] Siguier P, Gourbeyre E, Chandler M (2014). Bacterial insertion sequences: their genomic impact and diversity. FEMS Microbiol Rev.

[87] Tanaka KH, Frenette M, Charette SJ (2013). IS-mediated loss of virulence by *Aeromonas salmonicida*. Mob Genet Elements.

[88] Azegami K (2013). Suppressive effect of bacteriophage on bacterial palea browning of rice caused by *Pantoea ananatis*. J Gen Plant Pathol.

[89] Correa VR, Majerczak DR, Ammar E-D, Merighi M, Pratt RC (2012). The bacterium *Pantoea stewartii* uses two different type III secretion systems to colonize its plant host and insect vector. Appl Environ Microbiol.

[90] Coulthurst S (2019). The Type VI secretion system: a versatile bacterial weapon. Microbiology.

