# Supplementary material for: Characterization of Pantoea ananatis from rice planthoppers reveals a clade of rice-associated P. ananatis undergoing genome reduction
Source: Microb Genom. 2022 Dec 5;8(12):mgen000907. doi: 10.1099/mgen.0.000907 (PMC9837560; doi:10.1099/mgen.0.000907)
Supplement: Supplementary material 1 [file mgen-8-907-s001.pdf]

Supporting Figures

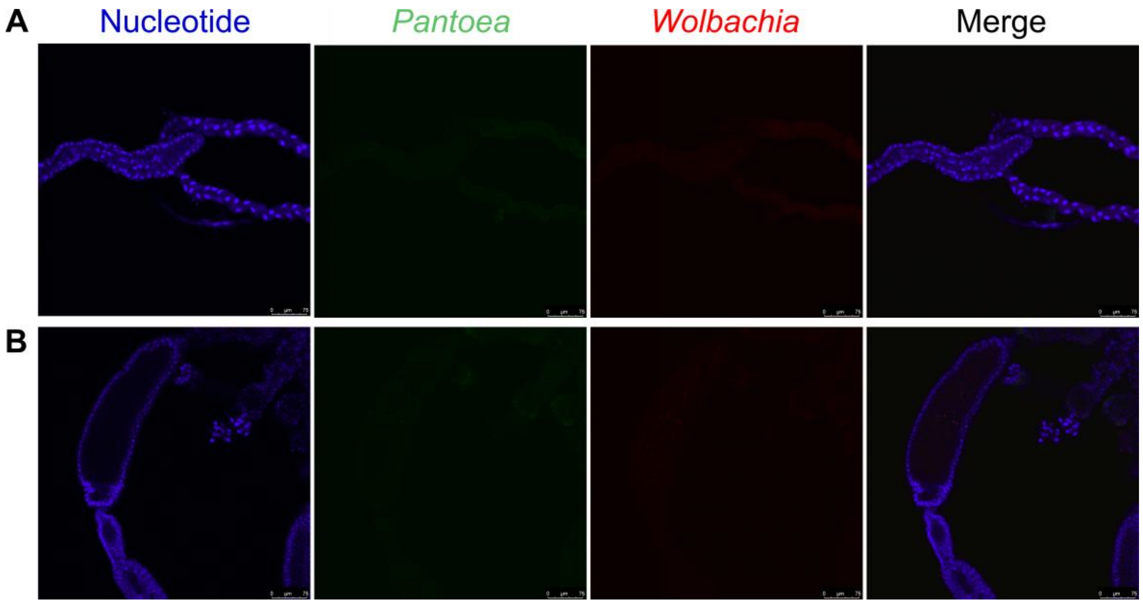

Figure S1. Confocal figures of the midgut (A) and ovary (B) of *L. striatellus*. Negative controls of the (A) midgut and (B) ovary tissues. The tissues were labeled with the nuclear DNA stain (DAPI, blue). No *Pantoea*-specific probe (FAM, green) or *Wolbachia*-specific probes (rhodamine, red) was added in these samples.

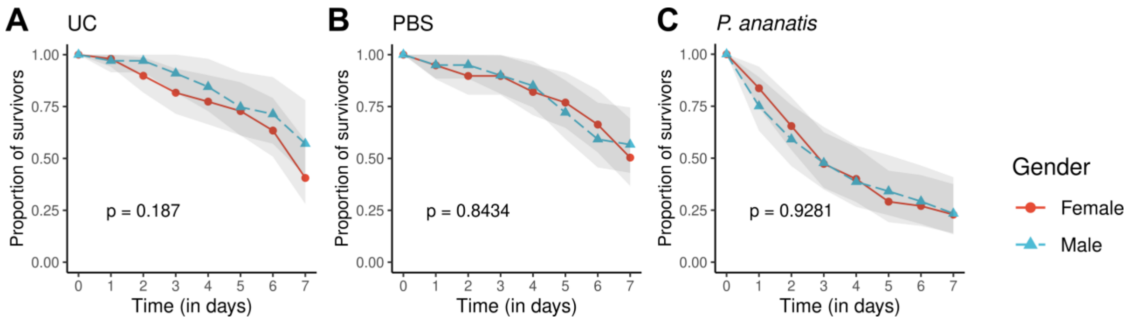

Figure S2. Survival of *L. striatellus* planthoppers after systemic infection. Survival curves represent the average percent survival and grey ribbons represent a 95% confidence interval. The p-values on each figure indicate the significant difference levels between the females and the males. UC, unchallenged control. *P. ananatis*, infection of *P. ananatis* with $O_{D600} = 1$.

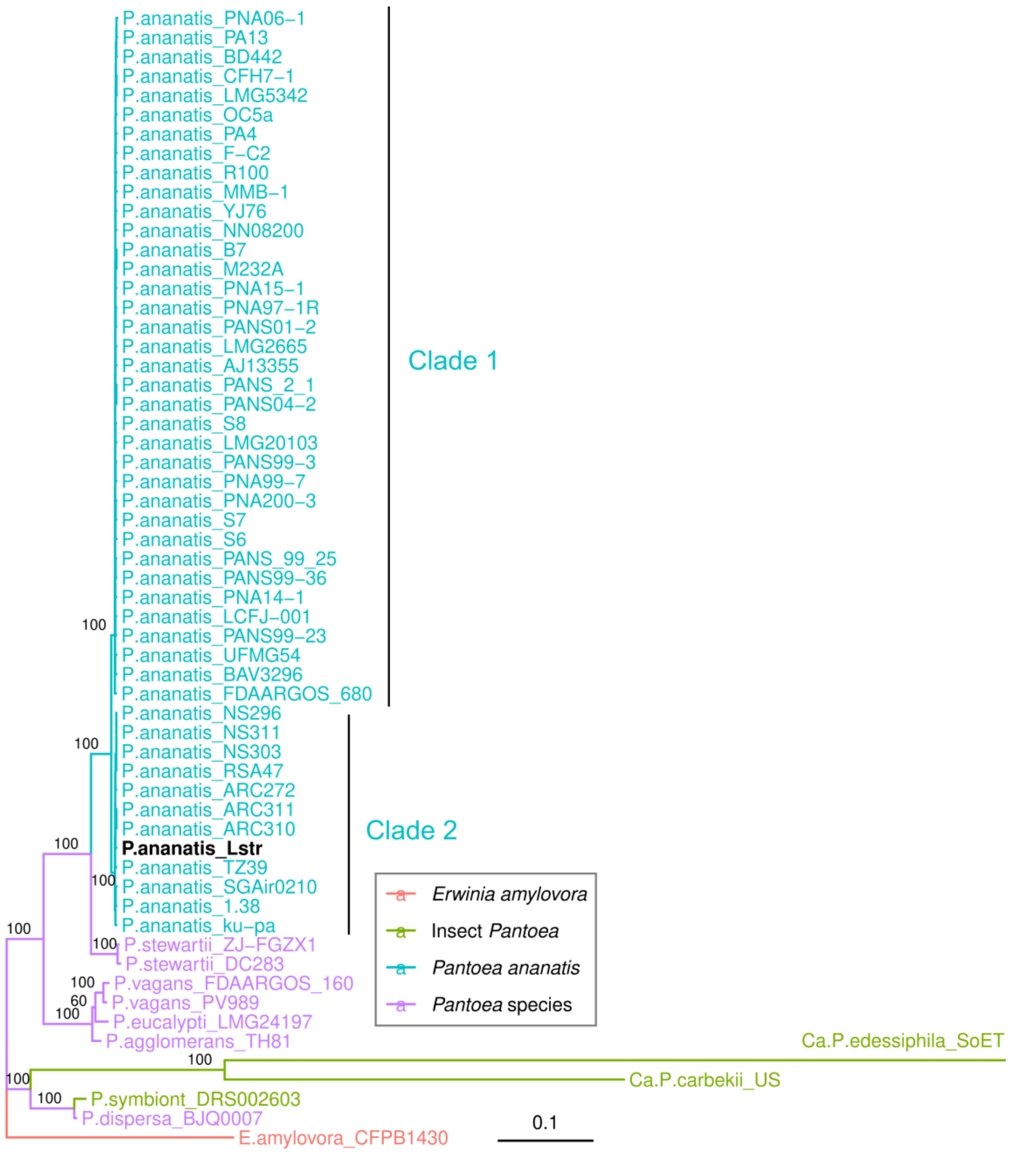

Figure S3. Phylogenomic analysis of *Pantoea* species.

The Maximum Likelihood (ML) tree was calculated with a concatenated protein sequence of single copy ortholog sequences (402176 aa) using a LG+R10 model. The categories of bacteria are color coded as shown on the branches. Bootstrap values are indicated at the respective nodes. The scale bar represents the average number of substitutions per site. Accession numbers of sequences in this tree were listed in Table S1.

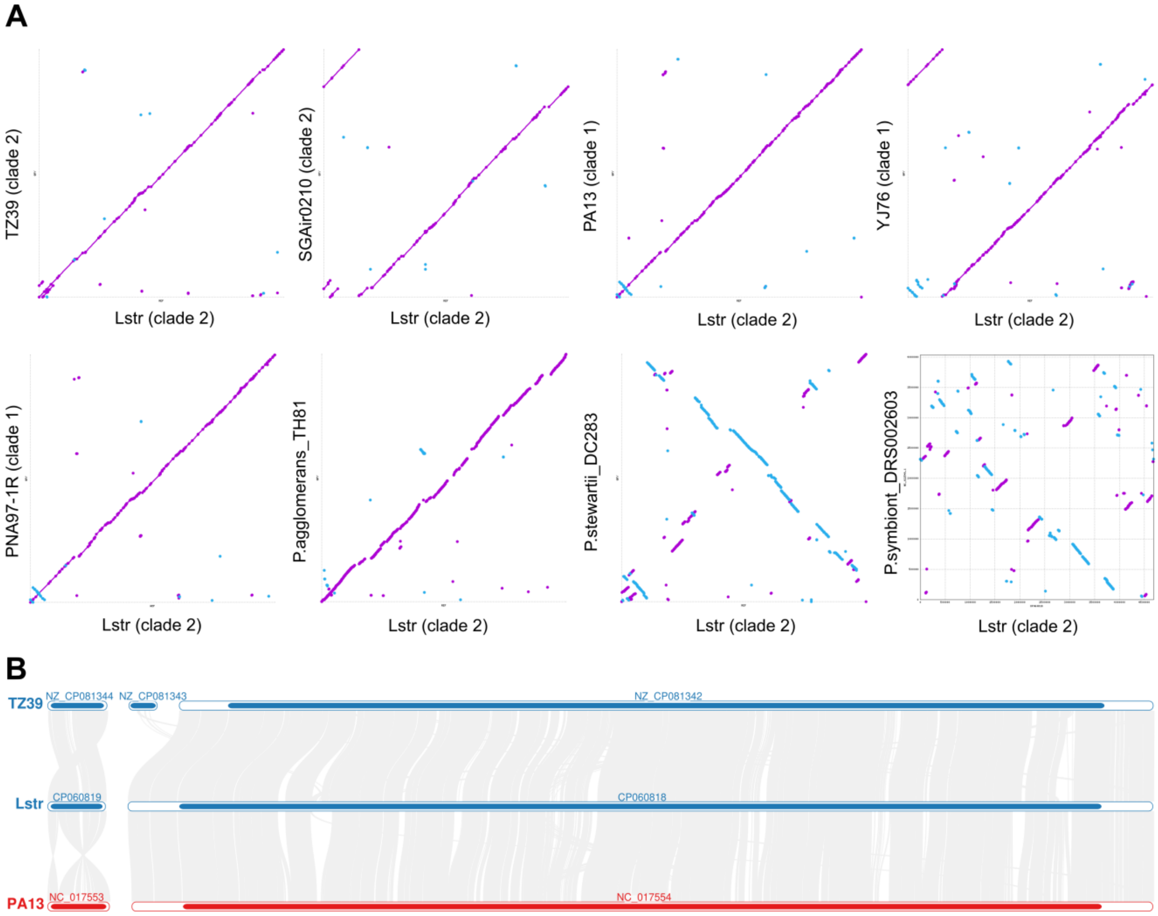

Figure S4. Syntenic conversation between *P. ananatis* Lstr and other *P. ananatis* genomes.

(A) Mummer plots. Dots and lines represent unique genomic sequence matches, purple for a forward match and blue for a reverse match (invasions). Numbers along the axes represent genome coordinates. The *P. ananatis* strain name and clade name are shown on axes. (B) Genomic alignments between rice-associated *P. ananatis* (TZ39, Lstr and PA13). Blue color indicates clade 2 and the red color clade 1. Gray wedges in the background highlight conserved synteny blocks with more than 10 gene pairs.

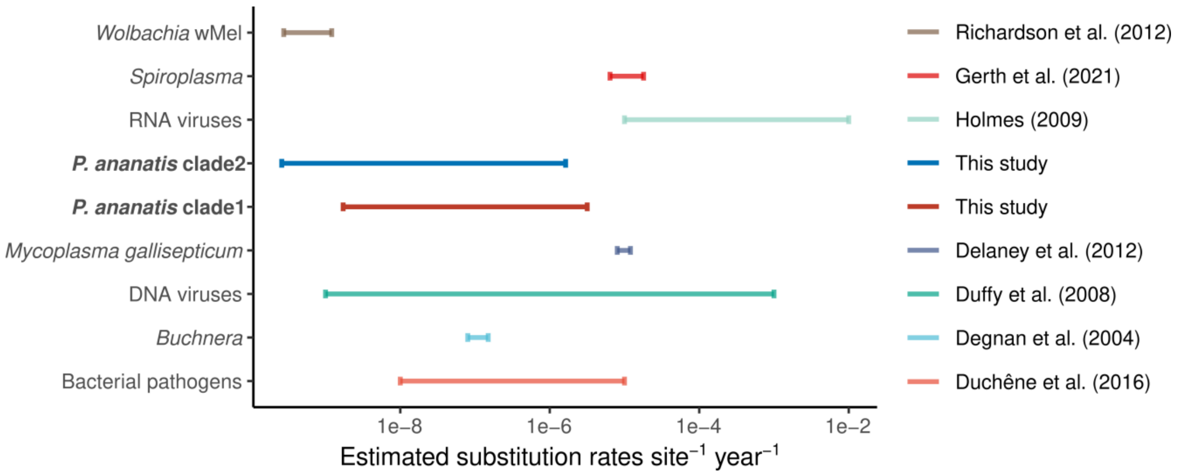

Figure S5. Comparison of estimated evolutionary rates across various microbes. Estimates obtained in this study are highlighted in bold. *Wolbachia w*Mel, genome-wide data extracted from Richardson et al [2]. *Spiroplasma*, estimated substitution rates from Gerth et al [3]. RNA viruses, range of RNA virus substitution rates from Holmes [4]. *Mycoplasma gallisepticu*, genome-wide rate estimated from Delaney et al [5]. DNA viruses, estimates as summarized by Duffy et al. [6], including ssDNA viruses and dsDNA viruses. *Buchnera*, genome-wide rate estimated from Moran [7]. Bacterial pathogens, approximate range of evolutionary rates estimated from genome-wide data of 16 bacterial pathogens (*Acinetobacter baumannii, Bordetella pertussis, Enterococcus faecium, Klebsiella pneumoniae, Mycobacterium leprae, Mycobacterium tuberculosis, Neisseria meningitidis, Pseudomonas aeruginosa, Salmonella enterica, Shigella dysenteriae, Shigella sonnei, Staphylococcus aureus, Streptococcus pneumoniae, Streptococcus pyogenes, Vibrio cholerae, Yersinia pestis*) as determined by Duchêne et al. [8].

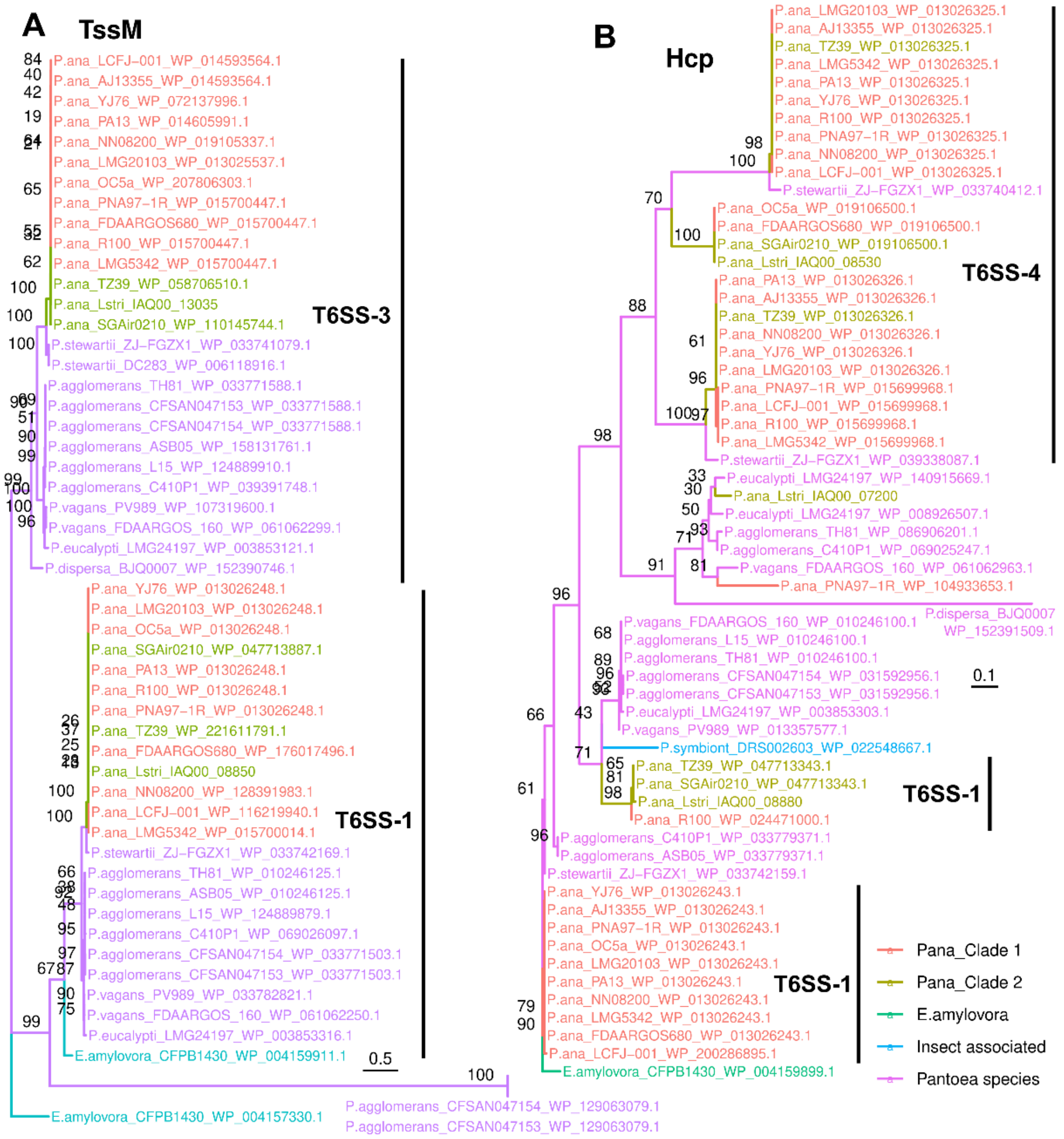

Figure S6. Phylogeny of (A) TssM and (B) Hcp.

The ML tree was constructed using a LG+F+G4 and LG+G4 substitution model for TssM and Hcp, respectively. The names of bacterial strains and the corresponding NCBI accession numbers of protein sequences are shown. Bootstrap values are indicated at the respective node. The scale bar represents the average number of substitutions per site.

Supplementary references

1. **Shalom G, Shaw JG, Thomas MS**. In vivo expression technology identifies a type VI secretion system locus in *Burkholderia pseudomallei* that is induced upon invasion of macrophages. *Microbiology* 2007;153(8):2689-2699.
2. **Richardson MF, Weinert LA, Welch JJ, Linheiro RS, Magwire MM et al.** Population genomics of the *Wolbachia* endosymbiont in *Drosophila melanogaster*. *PLoS Genet* 2012;8(12):e1003129.
3. **Gerth M, Martinez-Montoya H, Ramirez P, Masson F, Griffin JS et al.** Rapid molecular evolution of *Spiroplasma* symbionts of *Drosophila*. *Microbial Genomics* 2021;7(2).
4. **Holmes EC**. *The evolution and emergence of RNA Viruses*. New York: Oxford University Press; 2009.
5. **Delaney NF, Balenger S, Bonneaud C, Marx CJ, Hill GE et al.** Ultrafast evolution and loss of CRISPRs following a host shift in a novel wildlife pathogen, *Mycoplasma gallisepticum*. *PLoS Genet* 2012;8(2):e1002511.
6. **Duffy S, Shackelton LA, Holmes EC**. Rates of evolutionary change in viruses: patterns and determinants. *Nat Rev Genet* 2008;9(4):267-276.
7. **Moran NA, McLaughlin HJ, Sorek R**. The dynamics and time scale of ongoing genomic erosion in symbiotic bacteria. *Science* 2009;323(5912):379-382.
8. **Duchêne S, Holt KE, Weill F-X, Le Hello S, Hawkey J et al.** Genome-scale rates of evolutionary change in bacteria. *Microbial Genomics* 2016;2(11).

Supporting Tables

Table S1 Information of bacterial genomes for phylogenomic analysis.xlsx

Table S2 Genomes for evolutionary rate analysis.xlsx

Table S3 Genome-wide average nucleotide identity (ANI) between *P. ananatis* Lstr and other *Pantoea ananatis*.xlsx

Table S4 PhiSpy annotated prophage of *P. ananatis* Lstr.xlsx

Table S5 Pana. Lstr.gtf.annotation.xlsx

Table S6 Summary of IS families.xlsx

Table S7 Summary of Orthogroups COG.xlsx

Table S8 Summary of T3SS proteins in *Pantoea* genomes.xlsx
